# Native Mass Spectrometry Reveals Binding Modes of the Tumor Suppressor Protein p53 to Different DNA Response Elements

**DOI:** 10.1021/jasms.6c00066

**Published:** 2026-05-05

**Authors:** Erik Siefke, Christian Arlt, Andrea Sinz

**Affiliations:** † Department of Pharmaceutical Chemistry and Bioanalytics, 9176 Martin Luther University Halle-Wittenberg, 06120 Halle (Saale), Germany; ‡ Center for Structural Mass Spectrometry, Martin Luther University Halle-Wittenberg, 06120 Halle (Saale), Germany

**Keywords:** native, MS, p21, DNA, DNA-RE, binding, tetrameric, p53, isolated, p53:DNA, 20-bp

## Abstract

We used native mass spectrometry (MS) to investigate how the architecture of the p21 DNA response element (DNA-RE) controls the binding mode of the tumor suppressor protein p53. We analyzed tetrameric full-length wild-type p53 and compared its DNA binding behavior with a dimeric variant, p53_L344A_. In total, 37 DNA constructs derived from p21 DNA-RE were examined via native MS, including full-site sequences, isolated half-sites, variants differing in length and composition, and random DNA sequences. The aim was to define the minimal DNA requirements for p53 binding using native MS as a robust platform for comparative analysis of p53:DNA assemblies. Our results show that flanking regions of the full 20-bp DNA-RE have no influence on the initial formation of p53:DNA complexes. However, the complete 20-bp site is required for both p53_wild‑type_ and p53_L344A_ to bind to DNA as tetramers. Binding of a single dimer to an isolated half-site is insufficient for generating the stable tetrameric p53:DNA complex. These findings indicate that dimer-dimer interactions are crucial for stabilizing the tetrameric p53:DNA complex.

## Graphical abstract

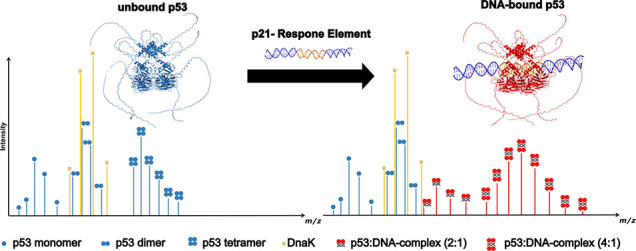

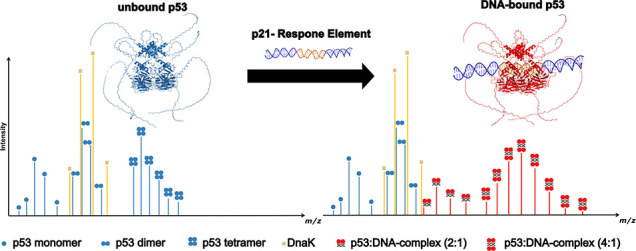

## Introduction

Native mass spectrometry (MS) is a powerful approach to characterize protein assemblies as it maintains noncovalent interactions.^1–3^ Protein-protein, protein-nucleic acid, and protein-small molecule interactions might be investigated in detail by native MS. Studying the interface between the tumor suppressor p53 and DNA can be efficiently addressed by native MS. P53 acts as a transcription factor that binds as a tetramer to its DNA response element (RE).^4^ Native MS is perfectly suited to study the interactions of p53, as it is able to resolve monomeric, dimeric, and tetrameric p53 species.^5^ Also, different stoichiometries of p53:DNA complexes have been successfully dissected for a 26-base pair (bp) DNA-RE by ion-mobility MS.^6^

P53 is commonly referred to as the “guardian of the genome” as it ensures the integrity of the genome.^7^ It influences cell cycle arrest, apoptosis, senescence, and DNA repair in response to cellular stress.^8,9^ Functionally, p53 operates as a sequence-specific transcription factor that recognizes its DNA-RE within promoters and enhancers of target genes.^10^ A p53 DNA-RE comprises two decameric half-sites with the consensus sequence RRRCWWGYYY (R: purine; Y: pyrimidine, W: adenine or thymine, C: cytosine, and g: guanine).^11,12^ Among the best-characterized downstream targets of p53 is p21, harboring multiple high-affinity full DNA-RE that mediate p53 binding.^13^ The p21 DNA-RE has therefore served as a paradigm for high-affinity recognition and cooperative assembly of p53 and its DNA recognition motifs.^13–15^

An important principle of p53 interaction with DNA is its dependence on p53’s oligomeric state. A p53 dimer can associate with a single half-site of DNA-RE (10 bp) and partially stabilize the interaction. The affinity of the p53 dimer:DNA complex is lower than that of the p53 tetramer:DNA.^16,17^ The p53 tetramer is the physiologically relevant DNA-binding unit: Each p53 dimer binds to one decameric half-site, occupying the full DNA-RE and enabling high-affinity binding, cooperative stabilization, and transcriptional control of targets such as p21.^8^

Here, we use native MS for the first time to analyze how the architecture of p21 DNA-RE controls the binding mode of p53. For our studies, we employ tetrameric p53 full-length wild-type species (p53_wild‑type_). In addition, we rely on a dimeric p53 variant to compare the DNA binding behavior of two defined oligomerization states of p53, tetramer, and dimer. To generate the dimeric p53 variant, a mutation was introduced at position 344, replacing a leucine with an alanine residue (p53_L344A_).^18^

We analyze a total of 37 DNA constructs derived from the p21 DNA-RE, including full-sites as well as isolated half-sites and DNA variants differing in length and composition in addition to random DNA sequences (Supporting Information, Table S1). The objective of this work is to determine the minimal DNA requirements for p53 binding using native MS as a robust platform for the comparative analysis of p53:DNA assemblies.

## Experimental Section

### Chemicals

All chemicals used were of the highest purity available and were obtained from Sigma-Aldrich. DNA response elements were purchased from Microsynth (Supporting Information, Table S1).

### Expression and Purification of p53

Wild-type tetrameric (p53_wild‑type_) and dimeric p53 (p53_L344A_) were expressed with N-terminal histidine-lipoyl domain tobacco etch virus tag (HLT) in *Escherichia coli* BL21 (DE3) cells, as previously described.^5,19^ Cells were harvested after incubation (18 °C, 16 h) and lysed by ultrasonication in 50 mM 2-[4–2-hydroxyethylpiperazine-1-yl]­ethanesulfonic acid (HEPES), 300 mM NaCl, 2.5 mM tris­(2-carboxyethyl)­phosphine (TCEP), and 20 mM imidazole, pH 7.4. The protein was purified by affinity chromatography on an FPLC system (ÄKTA Pure, GE Healthcare) using a 1 mL HisTrap HP column (Cytiva), followed by size-exclusion chromatography (SEC, Superdex 200 column, GE Healthcare) in 50 mM HEPES, 300 mM NaCl, 2.5 mM TCEP, 10% (v/v) glycerol, pH 7.4. After TEV cleavage at 4 °C, the SEC purification step was repeated overnight. The functionality of purified p53_wild‑type_ and p53_L344A_ was verified by native MS showing tetramerization and dimerization and by the DNA binding assay, as described previously.^5,19^

### Native MS

Proteins (p53_wild‑type_ and p53_L344A_, 9 μM each) were incubated with equimolar amounts of DNA-RE elements (Figure 1) at 4 °C overnight. The buffer system of p53_wild‑type_ and p53_L344A_ preparations was exchanged to 500 mM ammonium acetate, pH 6.8, using Amicon Ultra centrifugal filter units (0.5 mL; 3-, 10- or 30 kDa cutoff, Millipore). Native MS was carried out on a High-Mass Q-TOF 2 instrument (Waters Micromass/MS Vision) equipped with a nanoelectrospray ionization (ESI) source. The capillary voltage ranged from 1.2 to 1.4 kV, the sample cone voltage varied from 100 to 160 V, and the extraction cone voltage was set to 15 V. The acceleration voltage in the collision cell ranged from 30 to 90 V. The source pressure was adjusted to 10 mbar, and the pressure in the collision cell was adjusted to 1 × 10^–2^ to 2 × 10^–2^ mbar. Measurements were carried out using the MS profile mode for the quadrupole to guide ions within the *m*/*z* region of interest. Data were recalibrated with cesium iodide (CsI).

**Figure 1:**
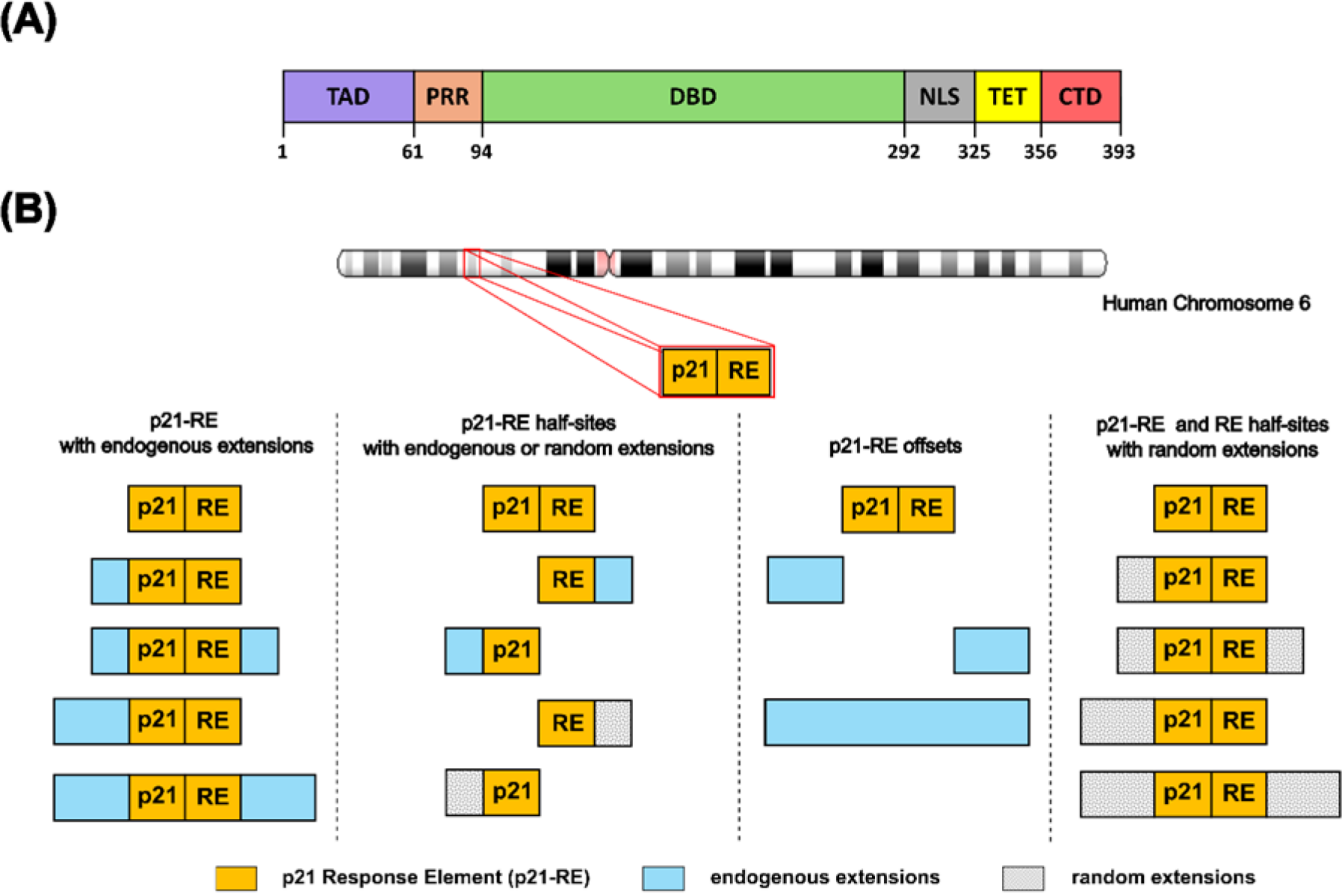
Overview of p53 domains and design of the p21 DNA-RE library. (A) Domain structure of p53. The domains are denoted as transactivation domain (TAD), proline rich region (PRR), DNA binding domain (DBD), nuclear localization signal (NLS), tetramerization domain (TET), C-terminal domain (CTD). (B) DNA library design, based on the p21 response element (p21-RE). Full-site DNA-RE, half-site DNA-RE as well as endogenous and random extensions are indicated.

## Results and Discussion

### Design of DNA Library for p53 Binding

The tumor suppressor protein p53 is a multidomain protein (Figure 1A) that acts as a transcription factor binding to the p21 DNA-RE (Figure 1B). For native MS studies, 37 different DNA-RE were generated to decipher the influence of p21-DNA-RE composition on the binding of p53_wild‑type_ and p53_L344A_. A DNA library was designed in the following manner, generating DNA-RE that contain

(i) both decameric half-sites plus endogenous extensions with variable lengths (10 to 40 bp),
(ii) only one or no decameric half-site,
(iii) both decameric half-sites plus random extensions (ATG repeats) with variable lengths (10 to 40 bp).

By this, we sought to investigate the influence of the regions flanking the p21 DNA-RE for binding to p53 dimeric and tetrameric variants and to check the ability of p53 to bind to only one decameric half-site of the DNA-RE or to other DNA sequences. A summary of all 37 DNA-RE used in this study is presented in the Supporting Information (Table S1).

### Native MS of p53:DNA Complexes

All 37 DNA-RE were investigated for their ability to form defined complexes with p53_wild‑type_ and p53_L344A_. Stoichiometries of the p53:DNA complexes created were determined by native MS. A comprehensive presentation of all mass spectra is presented in the Supporting Information (Figures S1–S37).

### Influence of Half-Site and Full-Site DNA-RE on the Formation of p53_wild‑type_DNA Complexes

Without the addition of DNA, signals of tetrameric, trimeric, dimeric, and monomeric p53 are visible for p53_wild‑type_ (Figure 2A) with charge states of +24 to +29 at *m*/*z* ∼6000 to 7500 (tetramer), +23 to +24 at *m*/*z* ∼5400 to 5800 (trimer), +17 to +20 at *m*/*z* ∼4250 to 5200 (dimer), and +11 to +15 at *m*/*z* 2750 to 4000 (monomer). Notably, non-DNA-bound tetrameric, dimeric, and monomeric species of p53_wild‑type_ were observed in all spectra, irrespective of DNA addition. We cannot rule out that these species might be caused by a partial dissociation of p53:DNA complexes in the gas phase; however, our results are in perfect agreement with previous native MS studies on p53.^5,6^ Upon incubation with full-site (20 bp) DNA-RE, the formation of specific p53:DNA complexes was observed, yielding signals between *m*/*z ∼*6400 and 8200 (+23 to +29 charge states) and ∼4900 to 5600 (+18 to +20 charge states) that correspond to 4:1 and 2:1 (p53:DNA) stoichiometries (Figure 2B). For a one half-site (10 bp) DNA-RE with an endogenous extension (10 bp), the exclusive formation of a 2:1 p53:DNA-complex is visible (Figure 2C).

**Figure 2:**
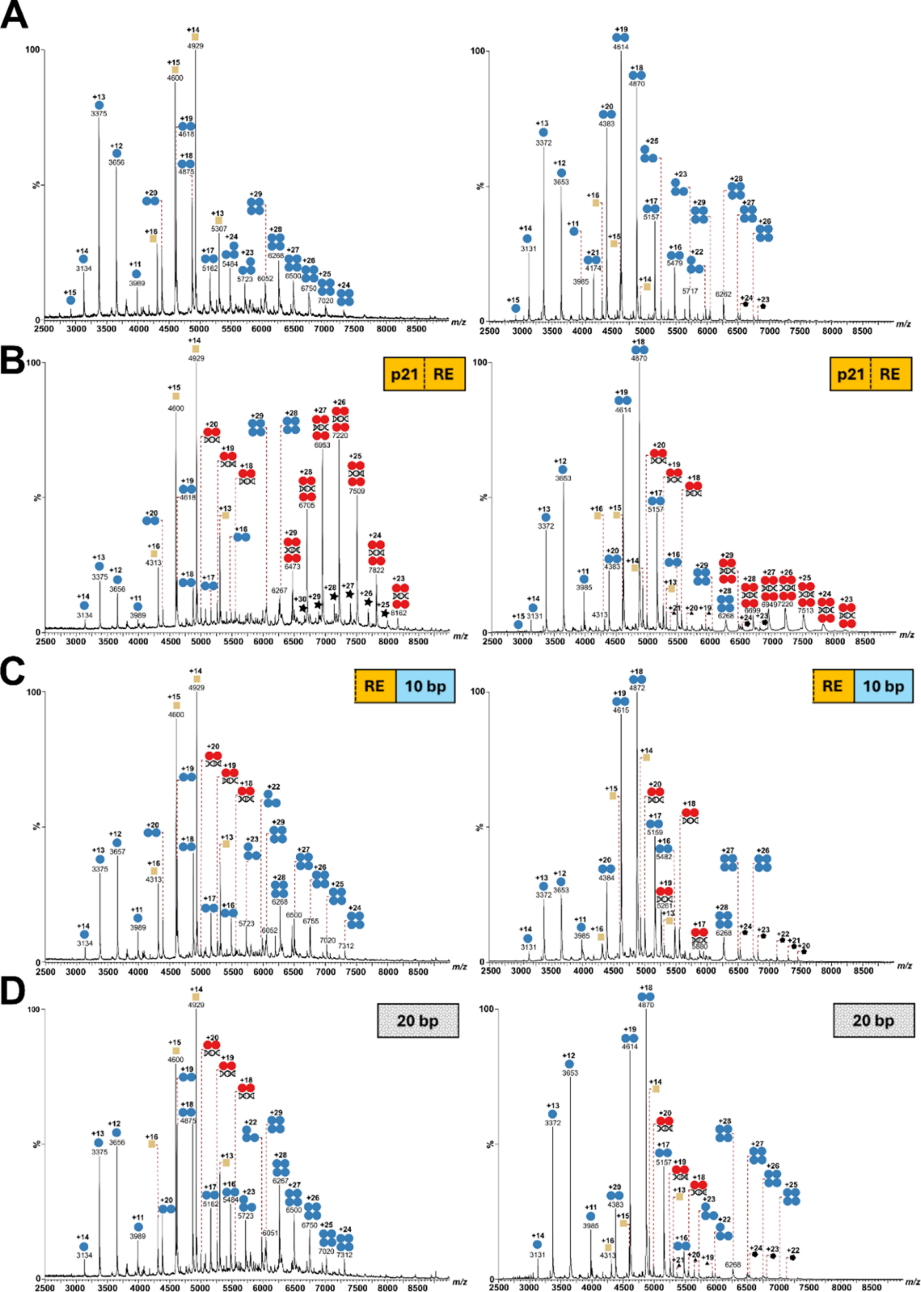
Native MS analysis of p53_wild‑type_ (left panel) and p53_L344A_ (right panel). (A) p53 without DNA. (B) p53 with full-site p21 DNA-RE (20 bp). (C) Half-site p21 DNA-RE (10 bp) plus 3′ endogenous DNA extension (10 bp). (D) Random DNA (20 bp). p53 (blue circle), p53:DNA complex (red circle), DnaK (yellow square), unknown 112 kDa species (black triangle), unknown 156 kDa species (black pentagon), and unknown 199 kDa species (black star).

To account for nonspecific DNA binding of p53_wild‑type_, a random 20-bp DNA strand was employed containing ATG repeats only (Figure 2D). Interestingly, for the random DNA stretch, an almost identical binding behavior was observed as for the DNA-RE-containing one half-site, with the formation of a 2:1 p53:DNA-complex. The fact that p53_wild‑type_ seems to bind to specific DNA-RE and nonspecific random DNA in a similar manner is at first puzzling. These results unambiguously indicate, however, that the full-site DNA sequence (20-bp) has to be present to induce the formation of a 4:1 (p53:DNA) complex. The presence of only a half-site motif in the DNA-RE is apparently insufficient to induce binding of the p53 tetramer to the DNA. This is the first time that the minimal requirements of DNA-RE binding to the tumor suppressor p53 are being studied in detail.

### Influence of Half-Site and Full-Site DNA-RE on the Formation of p53_L344A_:DNA Complexes

In the absence of DNA, the dimeric variant exhibits signals corresponding to monomeric, dimeric, and trimeric species, consistent with spectra observed for p53_wild‑type_ (Figure 2A). Unexpectedly, weak signals for tetrameric p53_L344A_ were also observed. This observation might be attributed to the protein concentration used in this study, which could promote the formation of gas-phase adducts in a concentration-dependent manner.^20^ As for p53_wild‑type_, non-DNA-bound species were detected for p53_L344A_ across all measurements (Figure 2). Upon incubation with the full-site (20 bp) DNA-RE, p53_L344A_ formed both 2:1 (+18 to +20 charge states at *m*/z ∼4900 to 5600) and 4:1 (+23 to +29 charge states at *m*/*z* ∼6400 to 8200) p53:DNA-complexes (Figure 2B), paralleling the results observed for p53_wild‑type_. In contrast, when incubated with a DNA-RE containing a single half-site (10 bp), together with an endogenous 10-bp extension or a random 20-bp DNA sequence, p53_L344A_ demonstrates a binding behavior similar to that of p53_wild‑type_, with only the 2:1 (p53:DNA) complex being visible (Figure 2C and D). These findings indicate that p53_L344A_ forms a 4:1 complex exclusively in the presence of full-site DNA-RE. Again, the presence of a single half-site is insufficient to promote the formation of a 4:1 (p53:DNA) complex, highlighting the necessity of full-site (20 bp) DNA-RE for generating the biologically active p53:DNA complex.

### Influence of DNA-RE Extensions on the Formation of p53_wild‑type_:DNA Complexes

To assess the impact of DNA-RE extensions on the p53_wild‑type_:DNA interaction, 10-bp extensions were added to the full-site DNA-RE on each site, namely, the 5′ and 3′ ends (Figure 1, Table S1). The same 10-bp extensions were added at the 5′ and 3′ ends of the half-site DNA-RE. All four extended DNA-REs were tested for their interactions with p53_wild‑type_ and p53_L344A_ (Figure 3).

**Figure 3:**
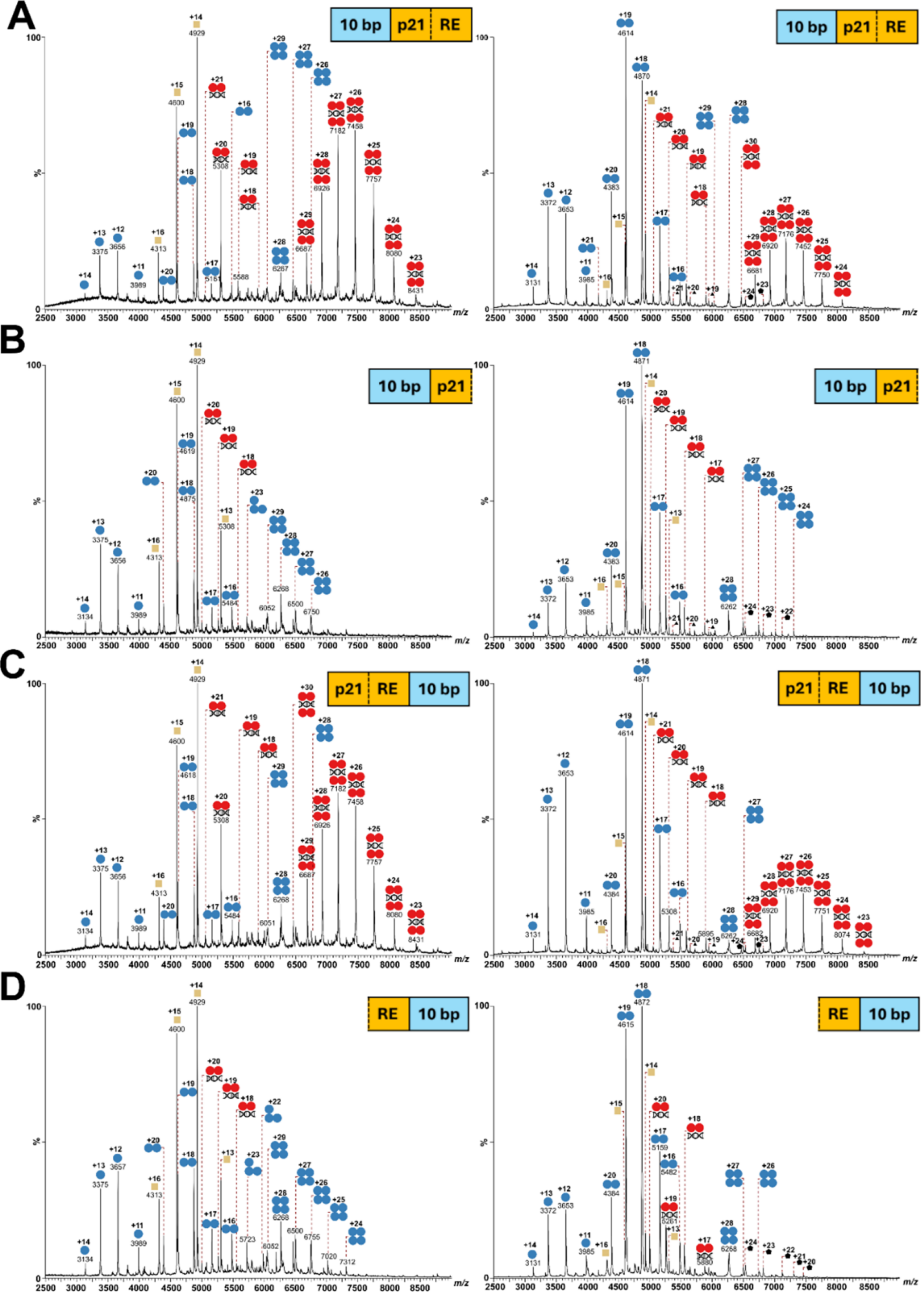
Native MS analysis of p53_wild‑type_ (left panel) and p53_L344A_ (right panel). (A) Full-site p21 DNA-RE (20 bp) plus 5′ endogenous DNA extension (10 bp). (B) Half-site p21 DNA-RE (10 bp) plus 5′ endogenous DNA extension (10 bp). (C) Full-site p21 DNA-RE (20 bp) plus 3′ endogenous DNA extension (10 bp). (D) Half-site p21 DNA-RE (10 bp) plus 3′ endogenous DNA extension (10 bp). p53 (blue circle), p53:DNA complex (red circle), DnaK (yellow square), unknown 112 kDa species (black triangle), and unknown 156 kDa species (black pentagon).

Incubation of p53_wild‑type_ with full-site DNA-RE showed signals for non-DNA-bound monomeric, dimeric, trimeric, and tetrameric p53, identical to what has been observed before (Figures 2 and 3). In addition, characteristic 2:1 and 4:1 (p53:DNA) stoichiometries were identified, with charge states ranging from +18 to +21 at *m*/*z* ∼5000 to 5900 (2:1 complex) and from +23 to +29 at *m*/*z* ∼6500 to 8500 (4:1 complex) (Figure 3A,C).

Incubation of p53_wild‑type_ with both half-site DNA-RE, extended with 10 bp at either end, again showed signals for non-DNA-bound monomeric, dimeric, trimeric, and tetrameric p53. In addition, formation of a 2:1 (p53:DNA) complex with +18 to +20 charge states at *m*/z ∼4900 to 5600 was observed (Figure 3B,D).

Similar observations were made for full-site and half-site DNA-RE-containing random extensions (Figures S19,S20,S25 and S26). These findings emphasize the necessity of a full-site (20 bp) DNA-RE for the assembly of the 4:1 (p53:DNA) complex. Extensions to the DNA-RE appear to have only a minimal or no impact at all on the initial formation of p53:DNA complexes.

### Influence of DNA-RE Extensions on the Formation of p53_L344A_:DNA Complexes

Comparable results were observed for p53_L344A_ following incubation with full-site DNA-RE (Figure 3A,C). The presence of 2:1 and 4:1 (p53_L344A_:DNA) complexes supports prior findings indicating that the full-site DNA-RE is required for the formation of a 4:1 (p53_L344A_:DNA) complex (Figure 3).

Incubation of p53_L344A_ with one half-site DNA-RE yielded results that are consistent with p53_wild‑type_: Only the 2:1 (p53_L344A_:DNA) complex (+18 to +20 at *m*/*z* ∼4900 to 5600) was detected (Figure 3B,D). An identical behavior was observed for DNA-RE-containing random extensions (Figure S19,S20,S25 and S26).

Again, these data highlight the requirement of a full-site (20 bp) DNA-RE for the successful assembly of the 4:1 (p53_L344A_:DNA) complex, which is identical to the situation of p53_wild‑type_. Notably, additional extensions to the DNA-RE have a minimal influence on the initial formation of p53:DNA complexes. Table 1 summarizes the stoichiometries observed for p53_wild type_ and p53_L344A_ complexes with DNA-RE.

**Table 1:**
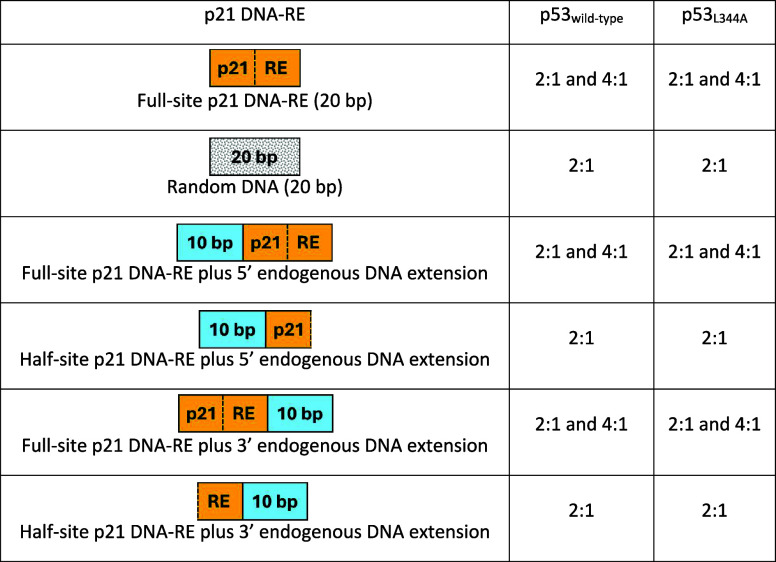
Stoichiometries of p53 Wild-type and p53_L344A_ Complexes with DNA-RE

## Conclusions

Our findings indicate that the flanking regions of the full-site (20-bp) DNA-RE have only a minor effect or no effect at all on the initial formation of p53:DNA complexes. Clearly, the full-site DNA-RE is required for an interaction of p53_wild‑type_ and p53_L344A_ with DNA as a tetramer (Figures 2 and 3). If one half-site DNA-RE is present, only the p53 dimer will bind. The dimer is apparently insufficient for the formation of a stable 4:1 (p53:DNA) complex, which seems to be the case for p53_wild‑type_ as well as for p53_L344A_. Our findings support the hypothesis that dimer-dimer interactions are required for the binding of the p53 tetramer to DNA.

## Supplementary Material



## Notes

Overview of p21 DNA-RE library and native mass spectra of p53_wild‑type_ and p53_L344A_ in the presence of different p21 RE-DNA strands (PDF)
