# Supplementary material for: Native Mass Spectrometry Reveals Binding Modes of the Tumor Suppressor Protein p53 to Different DNA Response Elements
Source: J Am Soc Mass Spectrom. 2026 May 5;37(6):1458–64. doi: 10.1021/jasms.6c00066 (PMC13237773; doi:10.1021/jasms.6c00066)
Supplement: Supplementary file 1 [file js6c00066_si_001.pdf]

Supporting Information

Native mass spectrometry reveals binding modes of the tumor suppressor protein p53 to different DNA response elements

Erik Siefke[1,2], Christian Arlt[1,2], Andrea Sinz[1,2,*]

[1] Department of Pharmaceutical Chemistry and Bioanalytics, Martin Luther University Halle-Wittenberg, 06120 Halle (Saale), Germany

[2] Center for Structural Mass Spectrometry, Martin Luther University Halle-Wittenberg, 06120 Halle (Saale), Germany

* Corresponding author:

Correspondence to:

Prof. Dr. Andrea Sinz
Center for Structural Mass Spectrometry
Martin Luther University Halle-Wittenberg
Halle (Saale), Germany
E-mail: andrea.sinz@pharmazie.uni-halle.de

Table S1. Overview of p21 DNA-RE library.

Name		Sequence 5´- 3´	NT	Pictogram
p21_A	TS	ATTGGCTTTCTGGCCGTCAGGAACATGTCCCAACATGTTG	40	20 bp p21 RE
	BS	CAACATGTTGGGACATGTTCCTGACGGCCAGAAAGCCAAT		
p21_B	TS	ATTGGCTTTCTGGCCGTCAG	20	20 bp
	BS	CTGACGGCCAGAAAGCCAAT		
p21_C	TS	TGGCCGTCAGGAACATGTCCCAACATGTTGAGCTCTGGCA	40	10 bp p21 RE 10 bp
	BS	TGCCAGAGCTCAACATGTTGGGACATGTTCCTGACGGCCA		
p21_D	TS	TGGCCGTCAGGAACATGTCCCAACATGTTG	30	10 bp p21 RE
	BS	CAACATGTTGGGACATGTTCCTGACGGCCA		
p21_E	TS	GAACATGTCCCAACATGTTGAGCTCTGGCATAGAAGAGGC	40	p21 RE 20 bp
	BS	GCCTCTTCTATGCCAGAGCTCAACATGTTGGGACATGTTC		
p21_F	TS	GAACATGTCCCAACATGTTGAGCTCTGGCA	30	p21 RE 10 bp
	BS	TGCCAGAGCTCAACATGTTGGGACATGTTC		
p21_G	TS	GAACATGTCCCAACATGTTG	20	p21 RE
	BS	CAACATGTTGGGACATGTTC		
p21_H	TS	CAACATGTTGAGCTCTGGCA	20	RE 10 bp
	BS	TGCCAGAGCTCAACATGTTG		
p21_I	TS	AGCTCTGGCATAGAAGAGGC	20	20 bp
	BS	GCCTCTTCTATGCCAGAGCT		
p21_J	TS	TGGCCGTCAGGAACATGTCC	20	10 bp p21
	BS	GGACATGTTCCTGACGGCCA		
p21_K	TS	ATGATGATGATCATCATCAT	20	20 bp
	BS	ATGATGATGATCATCATCAT		

p21_L	TS	GGCAGCAGGCTGTGGCTCTGATTGGCTTTCTGGCCG TCAGGAACATGTCCCAACATGTTG	60	40 bp p21 RE
	BS	CAACATGTTGGGACATGTTCCTGACGGCCAGAAAG CCAATCAGAGCCACAGCCTGCTGCC		
p21_M	TS	TGTGGCTCTGATTGGCTTTCTGGCCGTCAGGAACAT GTCCCAACATGTTG	50	30 bp p21 RE
	BS	CAACATGTTGGGACATGTTCCTGACGGCCAGAAAG CCAATCAGAGCCACA		
p21_N	TS	GAACATGTCCCAACATGTTGAGCTCTGGCATAGAAG AGGCTGGTGGCTAT	50	p21 RE 30 bp
	BS	ATAGCCACCAGCCTCTTCTATGCCAGAGCTCAACAT GTTGGGACATGTTC		
p21_O	TS	GAACATGTCCCAACATGTTGAGCTCTGGCATAGAAG AGGCTGGTGGCTATTTTGTCCTTG	50	p21 RE 40 bp
	BS	CAAGGACAAAATAGCCACCAGCCTCTTCTATGCCAG AGCTCAACATGTTGGGACATGTTC		
p21_P	TS	ATTGGCTTTCTGGCCGTCAGGAACATGTCCCAACAT GTTGAGCTCTGGCATAGAAGAGGC	60	20 bp p21 RE 20 bp
	BS	GCCTCTTCTATGCCAGAGCTCAACATGTTGGGACAT GTTCCTGACGGCCAGAAAGCCAAT		
p21_Q	TS	ATGATGATGATCATCATCATATGATGATGATCATCA TCATATGATGATGATCATCATCAT	60	60 bp
	BS	ATGATGATGATCATCATCATATGATGATGATCATCA TCATATGATGATGATCATCATCAT		
p21_R	TS	GAACCTGGGAGGCGGAGACTGCAGTGAGCTGAGA TTGTGCCACTGCTGACTTTGTCTCAA	60	60 bp
	BS	TTGAGACAAAGTCAGCAGTGGCACAATCTCAGCTCA CTGCAGTCTCCGCCTCCCAGGTTC		
p21_S	TS	CAACATGTTGTAGTTACGAT	20	RE 10 bp
	BS	ATCGTAACTACAACATGTTG		
p21_T	TS	CGATGCAGCAGAACATGTCC	20	10 bp p21
	BS	GGACATGTTCTGCTGCATCG		
p21_U	TS	CAACATGTTGTAGTTACGATAGCAGTGGAT	30	RE 20 bp
	BS	ATCCACTGCTATCGTAACTACAACATGTTG		
p21_V	TS	TGGGTCCTAGCGATGCAGCAGAACATGTCC	30	20 bp p21
	BS	GGACATGTTCTGCTGCATCGCTAGGACCCA		

p21_W	TS	CGATGCAGCAGAACATGTCCCAACATGTTGTAGTTACGAT	40	10 bp | p21 RE | 10 bp
	BS	ATCGTAACTACAACATGTTGGGACATGTTCTGCTGCATCG		
p21_X	TS	TGGGTCCTAGCGATGCAGCAGAACATGTCCCAACATGTTGTAGTTACGAT	50	20 bp | p21 RE | 10 bp
	BS	ATCGTAACTACAACATGTTGGGACATGTTCTGCTGCATCGCTAGGACCCA		
p21_Y	TS	GAACATGTCCCAACATGTTGTAGTTACGAT	30	p21 RE | 10 bp
	BS	ATCGTAACTACAACATGTTGGGACATGTTC		
p21_Z	TS	CGATGCAGCAGAACATGTCCCAACATGTTG	30	10 bp | p21 RE
	BS	CAACATGTTGGGACATGTTCTGCTGCATCG		
p21_1	TS	CGATGCAGCAGAACATGTCCCAACATGTTGTAGTTACGATAGCAGTGGAT	50	10 bp | p21 RE | 20 bp
	BS	ATCCACTGCTATCGTAACTACAACATGTTGGGACATGTTCTGCTGCATCG		
p21_2	TS	TGGGTCCTAGCGATGCAGCAGAACATGTCCCAACATGTTGTAGTTACGATAGCAGTGGAT	60	20 bp | p21 RE | 20 bp
	BS	ATCCACTGCTATCGTAACTACAACATGTTGGGACATGTTCTGCTGCATCGCTAGGACCCA		
p21_3	TS	GAACATGTCCCAACATGTTGTAGTTACGATAGCAGTGGAT	40	p21 RE | 20 bp
	BS	ATCCACTGCTATCGTAACTACAACATGTTGGGACATGTTC		
p21_4	TS	TGGGTCCTAGCGATGCAGCAGAACATGTCCCAACATGTTG	40	20 bp | p21 RE
	BS	CAACATGTTGGGACATGTTCTGCTGCATCGCTAGGACCCA		
p21_5	TS	TAGTTACGATAGCAGTGGAT	20	| 20 bp
	BS	ATCCACTGCTATCGTAACTA		
p21_6	TS	TGGGTCCTAGCGATGCAGCA	20	20 bp |
	BS	TGCTGCATCGCTAGGACCCA		
p21_7	TS	AGCTCTGGCATAGAAGAGGCGAACATGTCCCAACATGTTGATTGGCTTTCTGGCCGTCAG	60	20 bp | p21 RE | 20 bp
	BS	CTGACGGCCAGAAAGCCAATCAACATGTTGGGACATGTTCGCCTCTTCTATGCCAGAGCT		
p21_8	TS	ATGATGATGATCATCATCATGAACATGTCCCAACATGTTGATGATGATGATCATCATCAT	60	20 bp | p21 RE | 20 bp
	BS	ATGATGATGATCATCATCATCAACATGTTGGGACATGTTCATGATGATGATCATCATCAT		

p21_9	TS	GAACCTGGGAGGCGGAGACTGAACATGTCCCAACA TGTTGCACTGCTGACTTTGTCTCAA	60	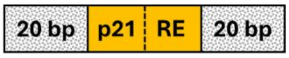
	BS	TTGAGACAAAGTCAGCAGTGCAACATGTTGGGACA TGTTCAGTCTCCGCCTCCCAGGTTC		
p21_10	TS	GGCAGCAGGCTGTGGCTCTGGAACATGTCCCAACA TGTTGAGCTCTGGCATAGAAGAGGC	60	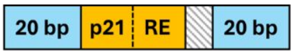
	BS	GCCTCTTCTATGCCAGAGCTCAACATGTTGGGACAT GTTCCAGAGCCACAGCCTGCTGCC		
p21_11	TS	ATTGGCTTTCTGGCCGTCAGGAACATGTCCCAACAT GTTGTGGTGGCTATTTTGTCCTTG	60	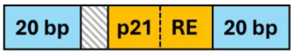
	BS	CAAGGACAAAATAGCCACCACAACATGTTGGGACA TGTTCCTGACGGCCAGAAAGCCAAT		

p21 Response Element (p21-RE) endogenous extensions repetitive extensions

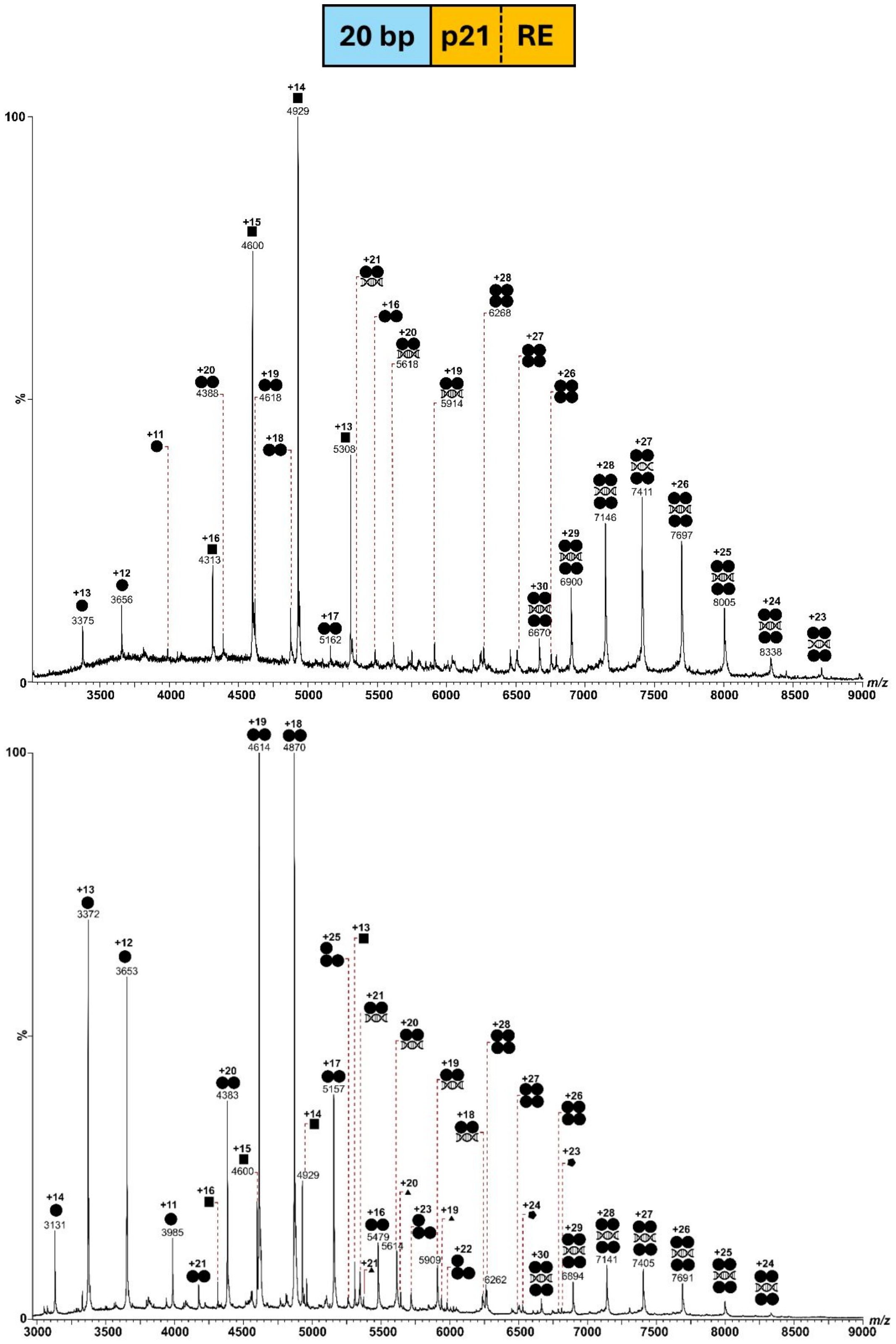

Figure S1. Native mass spectra of p53$_{\text{wild-type}}$ (upper spectrum) and p53$_{\text{L344A}}$ (lower spectrum) in the presence of DNA-RE p21_A. The DNA-RE is schematically depicted on top of the mass spectra. p53 (circle), p53:DNA complex (circle with DNA), DnaK (square), unknown 112 kDa species (triangle), unknown 156 kDa species (pentagon), unknown 199 kDa species (star).

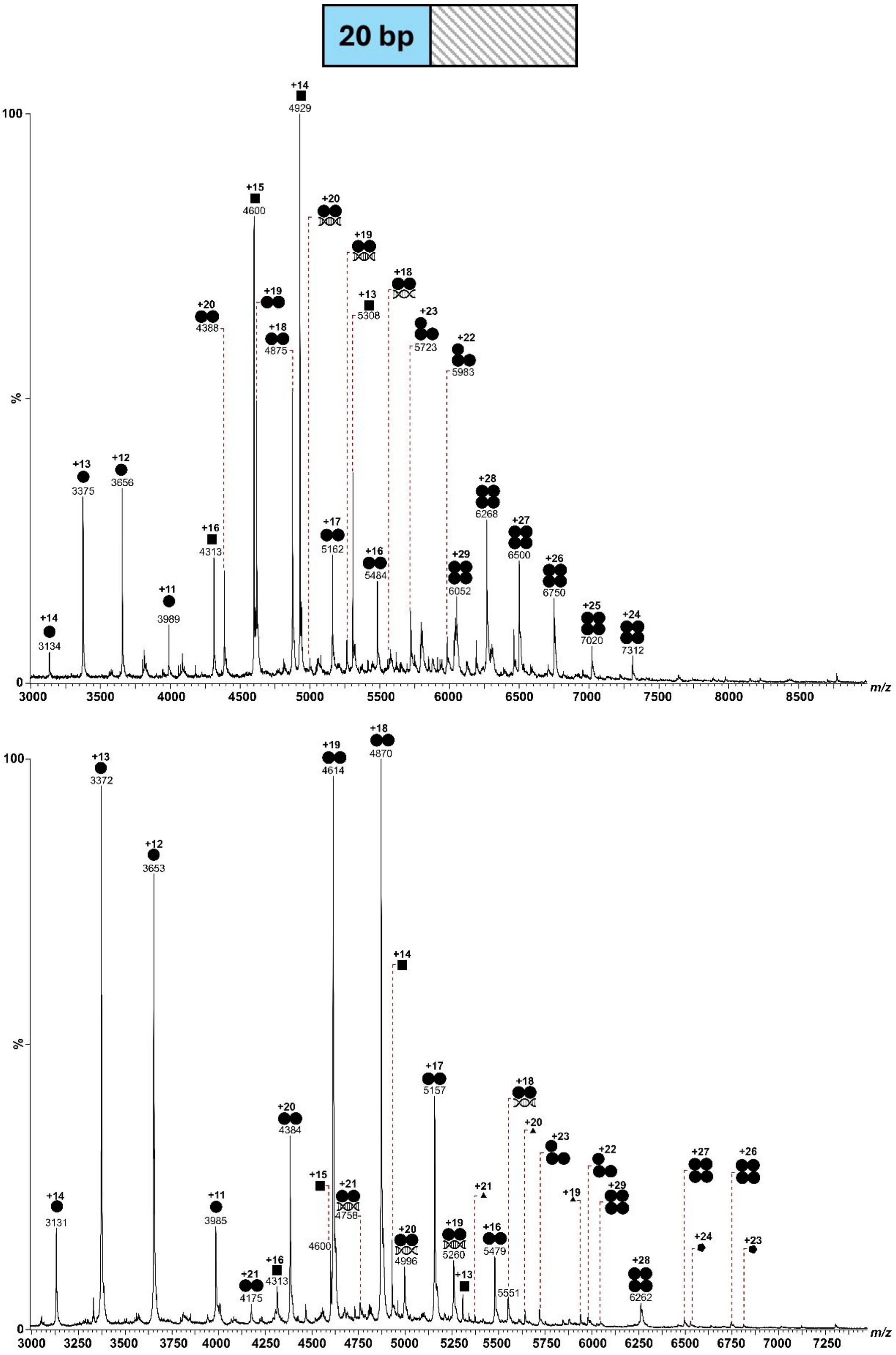

Figure S2. Native mass spectra of p53$_{wild\text{-}type}$ (upper spectrum) and p53$_{L344A}$ (lower spectrum) in the presence of DNA-RE p21_B. The DNA-RE is schematically depicted on top of the mass spectra. p53 (circle), p53:DNA-complex (circle with DNA), DnaK (square), unknown 112 kDa species (triangle), unknown 156 kDa species (pentagon), unknown 199 kDa species (star).

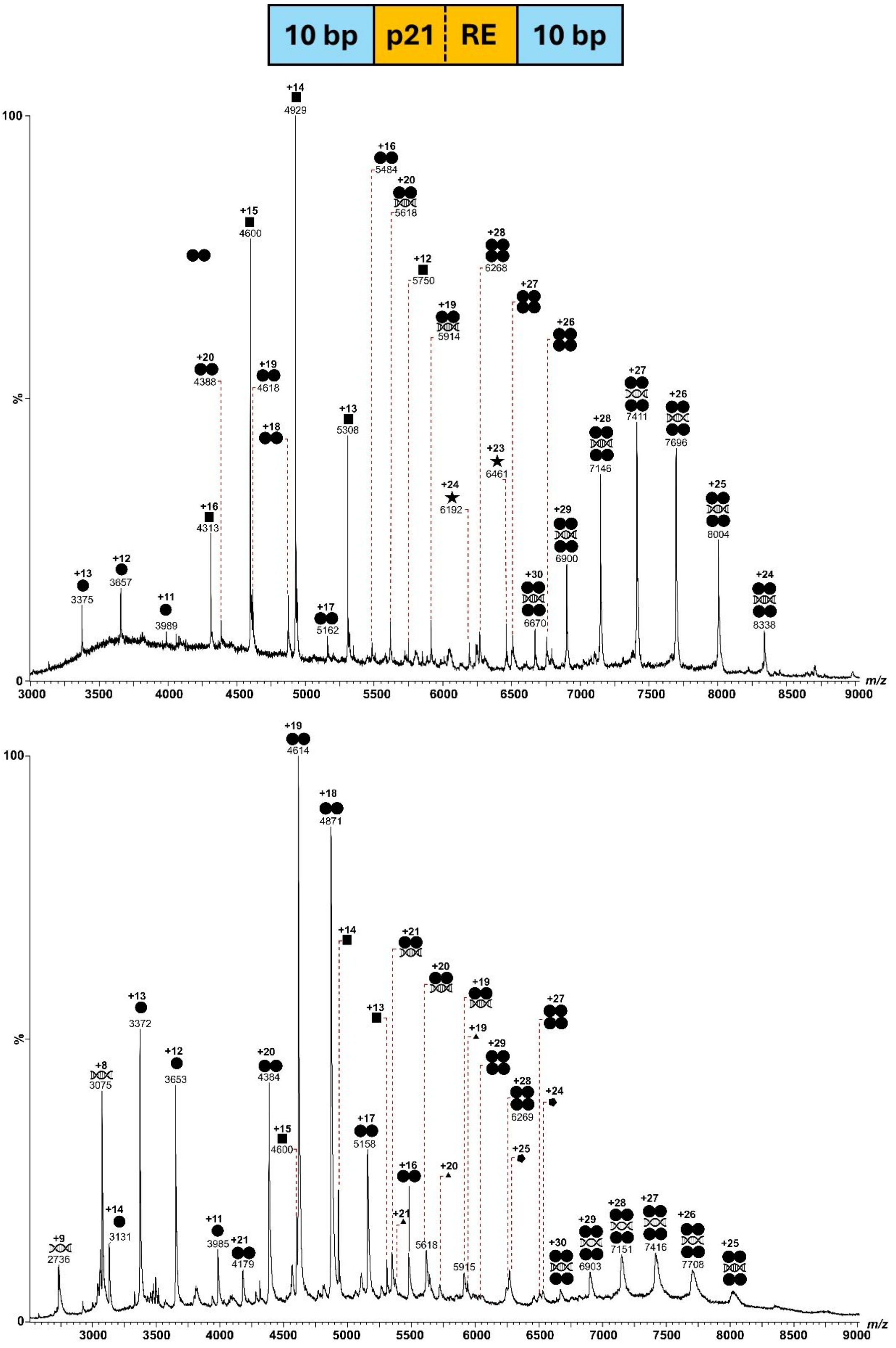

Figure S3. Native mass spectra of $p53_{wild\text{-}type}$ (upper spectrum) and $p53_{L344A}$ (lower spectrum) in the presence of DNA-RE p21_C. The DNA-RE is schematically depicted on top of the mass spectra. p53 (circle), p53:DNA-complex (circle with DNA), DnaK (square), unknown 112 kDa species (triangle), unknown 156 kDa species (pentagon), unknown 199 kDa species (star).

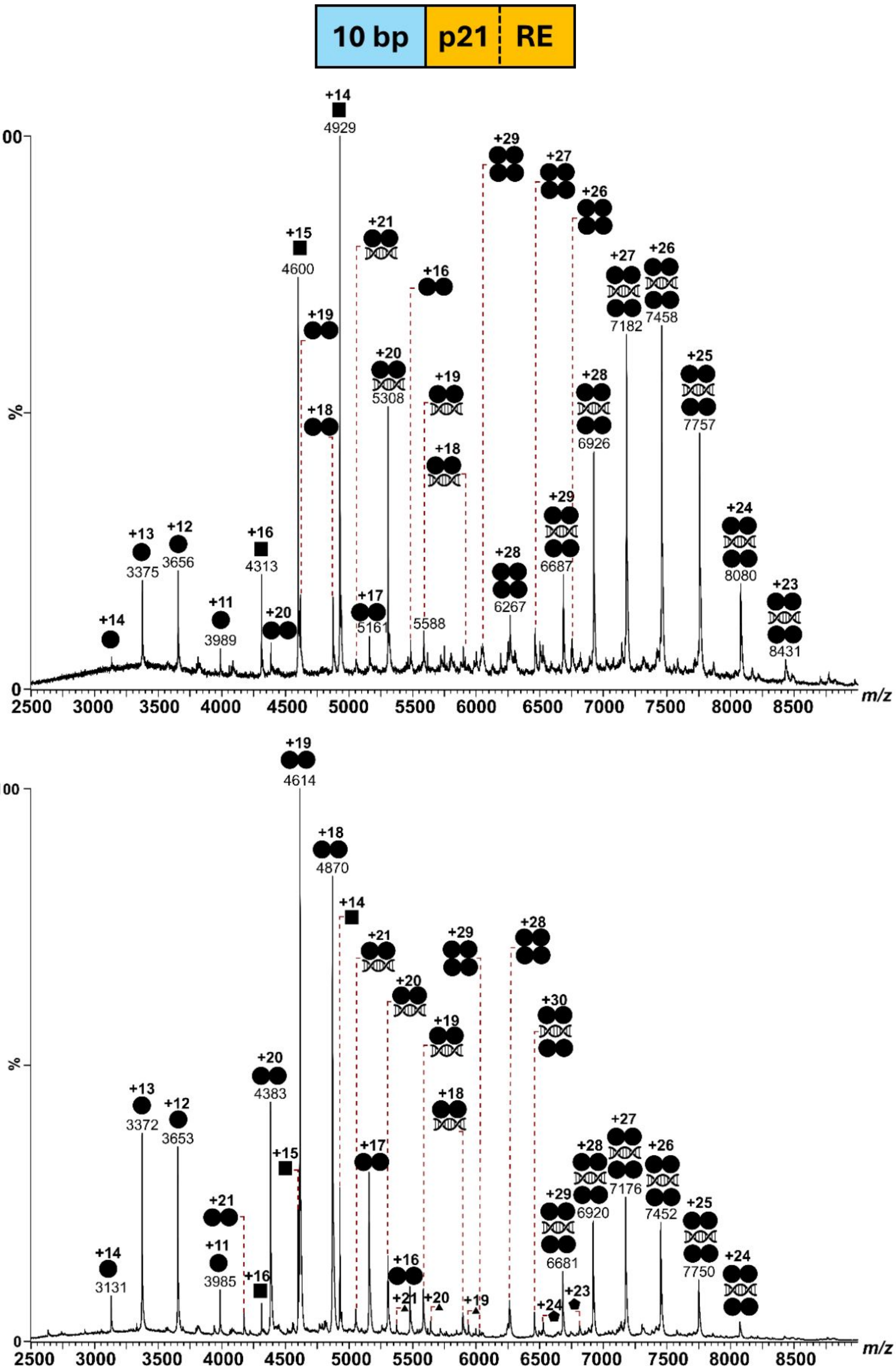

Figure S4. Native mass spectra of $p53_{wild\text{-}type}$ (upper spectrum) and $p53_{L344A}$ (lower spectrum) in the presence of DNA-RE p21_D. The DNA-RE is schematically depicted on top of the mass spectra. p53 (circle), p53:DNA-complex (circle with DNA), DnaK (square), unknown 112 kDa species (triangle), unknown 156 kDa species (pentagon), unknown 199 kDa species (star).

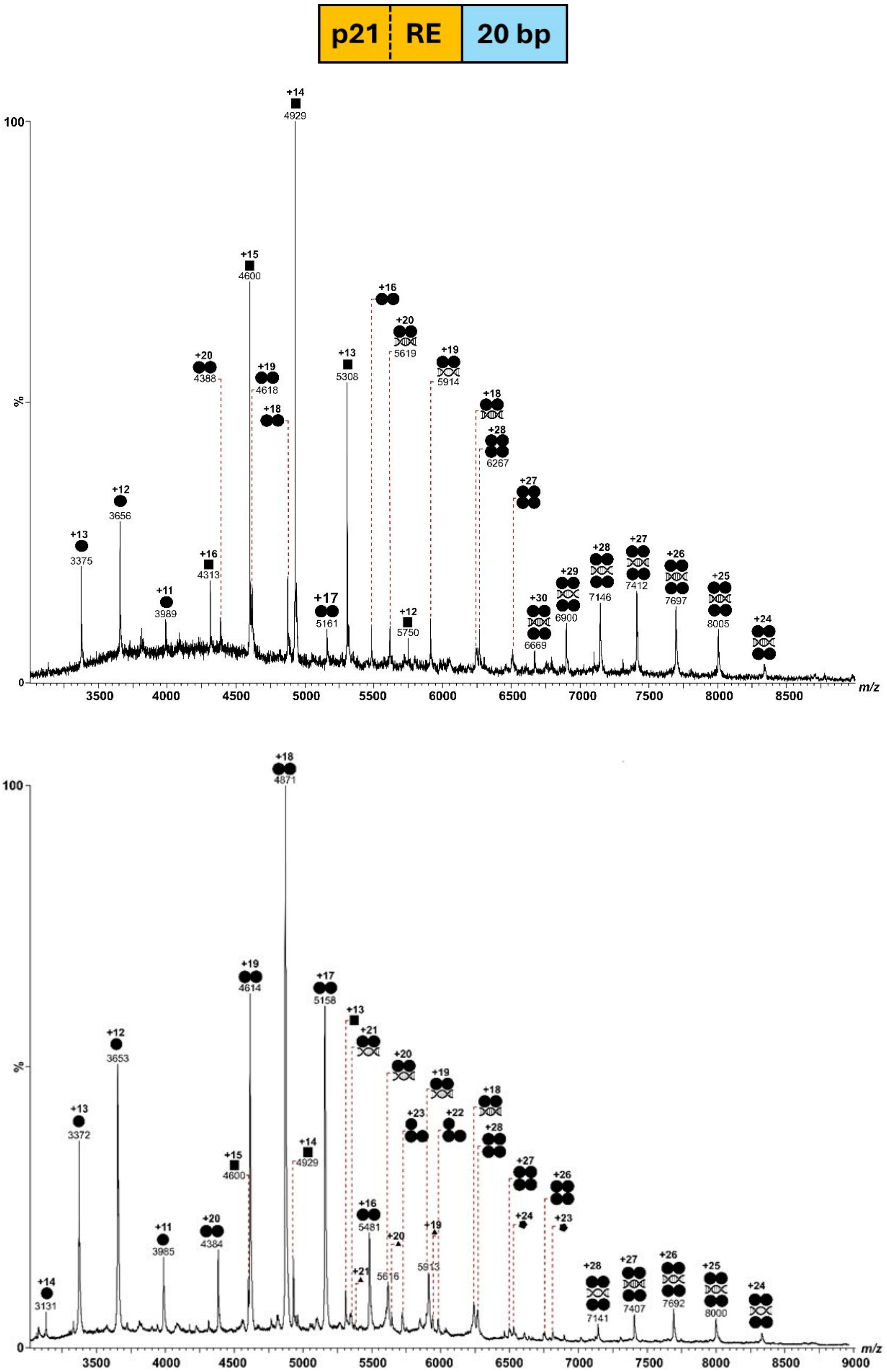

Figure S5. Native mass spectra of $p53_{wild\text{-}type}$ (upper spectrum) and $p53_{L344A}$ (lower spectrum) in the presence of DNA-RE p21_E. The DNA-RE is schematically depicted on top of the mass spectra. p53 (circle), p53:DNA-complex (circle with DNA), DnaK (square), unknown 112 kDa species (triangle), unknown 156 kDa species (pentagon), unknown 199 kDa species (star).

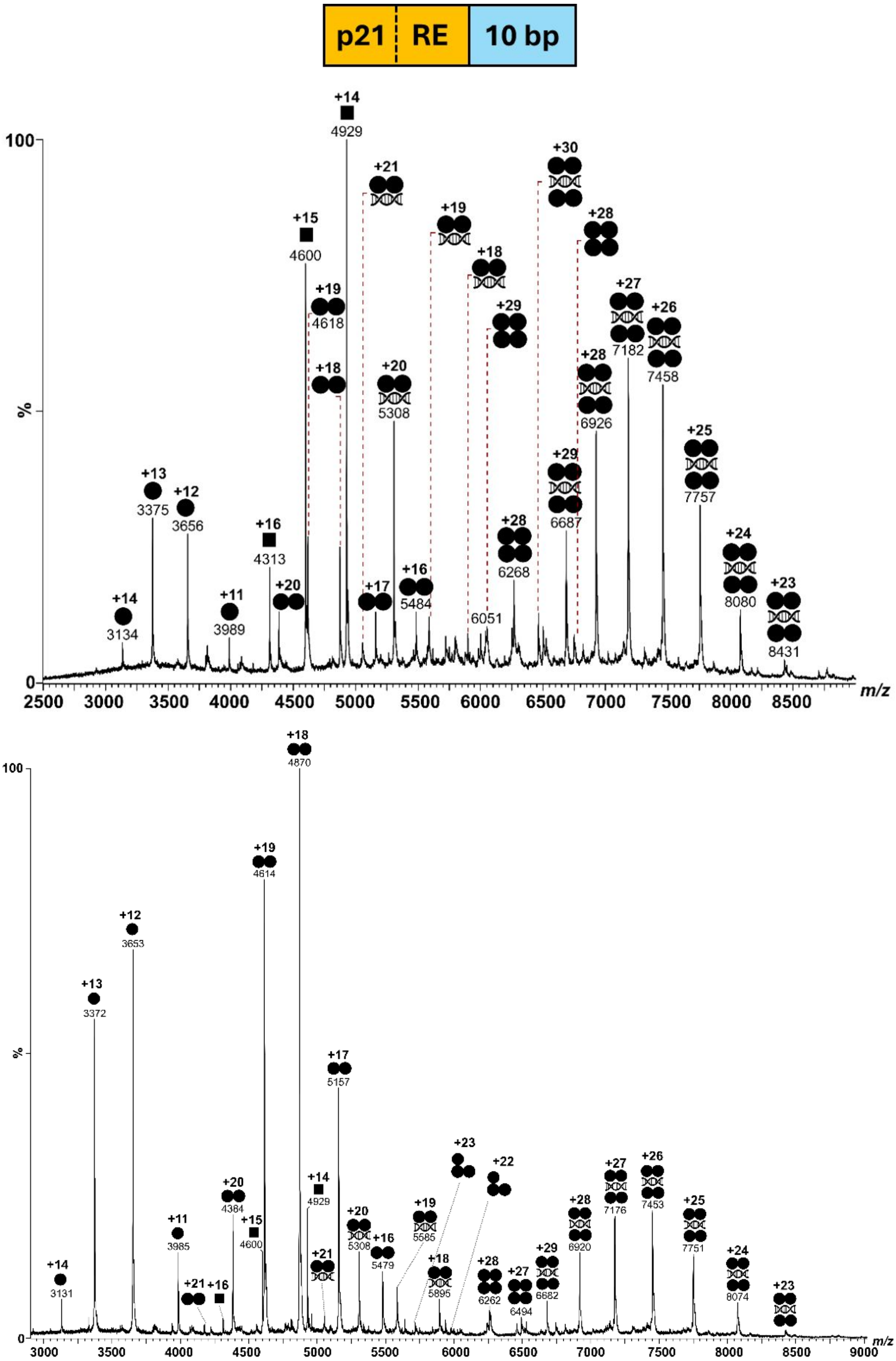

Figure S6. Native mass spectra of p53$_{wild\text{-}type}$ (upper spectrum) and p53$_{L344A}$ (lower spectrum) in the presence of DNA-RE p21_F. The DNA-RE is schematically depicted on top of the mass spectra. p53 (circle), p53:DNA-complex (circle with DNA), DnaK (square), unknown 112 kDa species (triangle), unknown 156 kDa species (pentagon), unknown 199 kDa species (star).

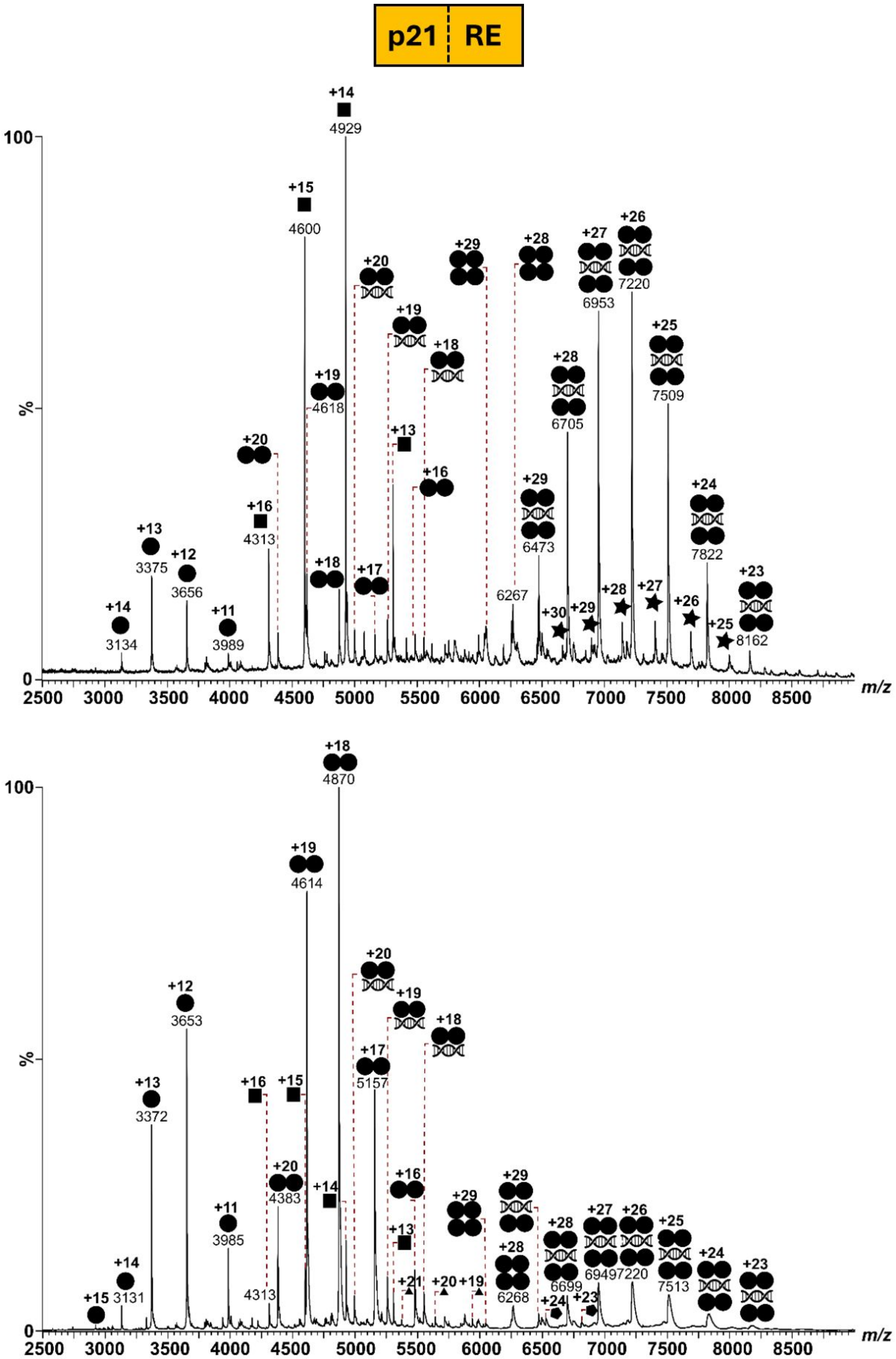

Figure S7. Native mass spectra of $p53_{wild\text{-}type}$ (upper spectrum) and $p53_{L344A}$ (lower spectrum) in the presence of DNA-RE p21_G. The DNA-RE is schematically depicted on top of the mass spectra. p53 (circle), p53:DNA-complex (circle with DNA), DnaK (square), unknown 112 kDa species (triangle), unknown 156 kDa species (pentagon), unknown 199 kDa species (star).

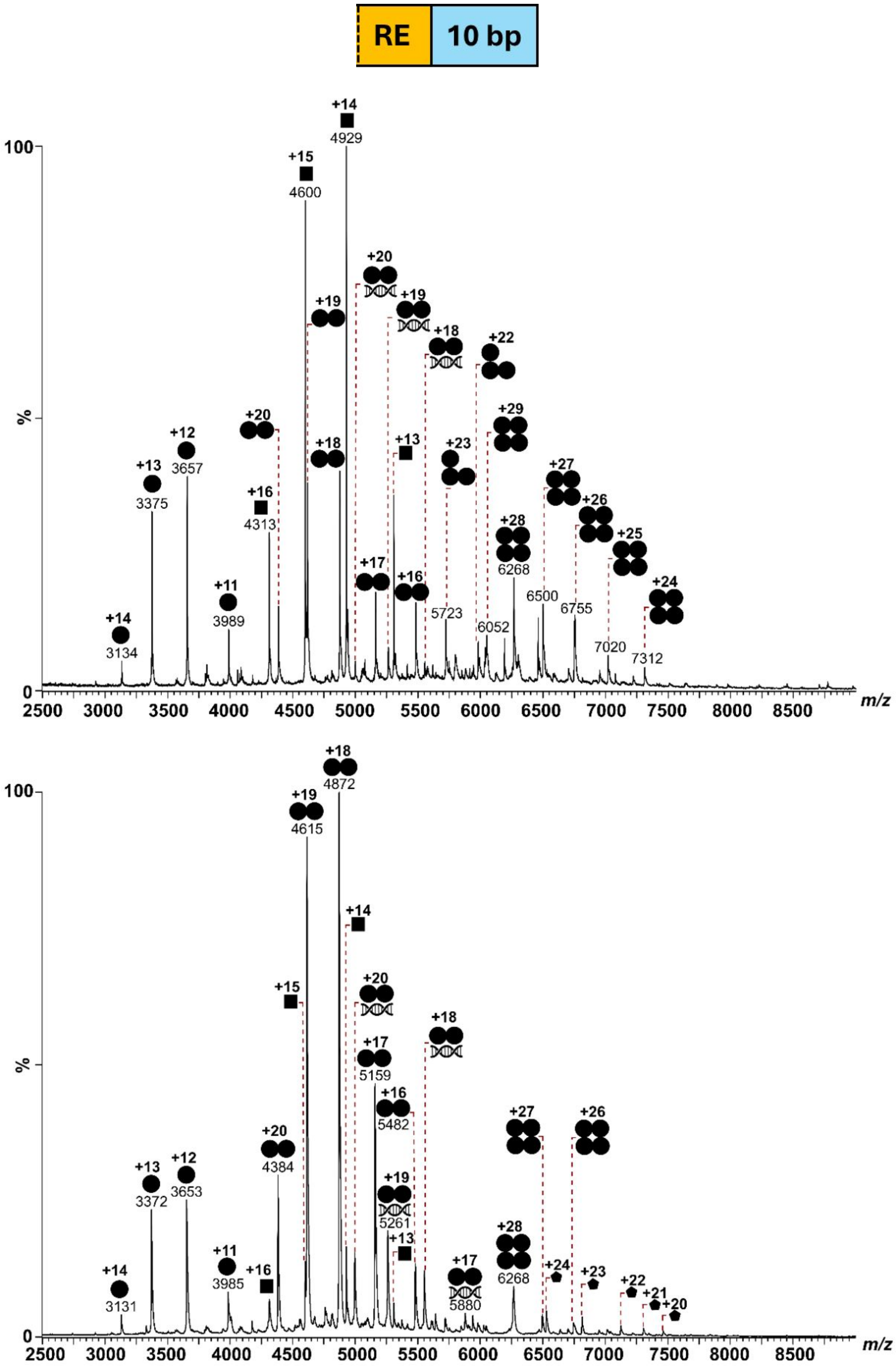

Figure S8. Native mass spectra of $p53_{wild\text{-}type}$ (upper spectrum) and $p53_{L344A}$ (lower spectrum) in the presence of DNA-RE p21_H. The DNA-RE is schematically depicted on top of the mass spectra. p53 (circle), p53:DNA-complex (circle with DNA), DnaK (square), unknown 112 kDa species (triangle), unknown 156 kDa species (pentagon), unknown 199 kDa species (star).

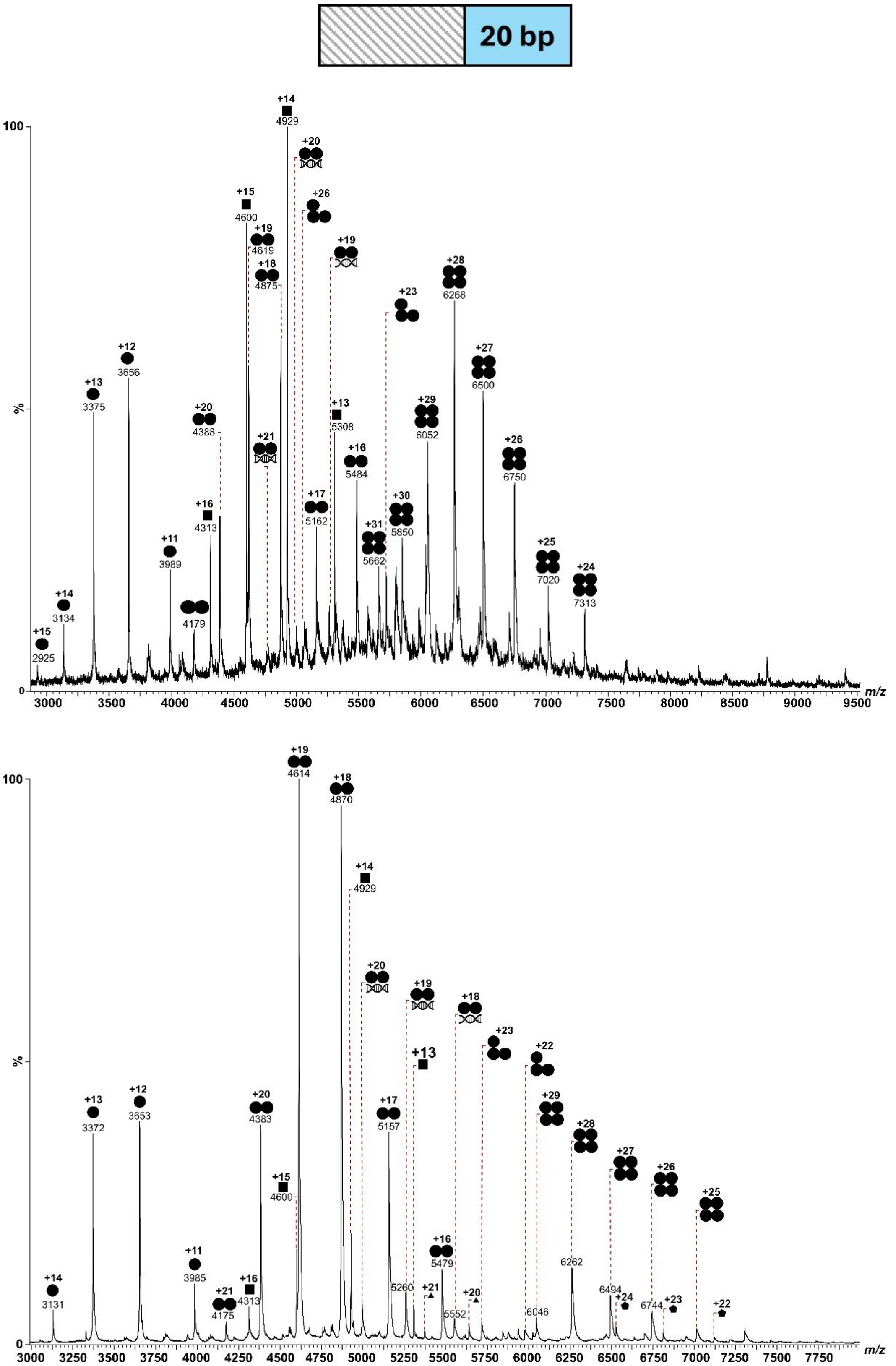

Figure S9. Native mass spectra of $p53_{wild\text{-}type}$ (upper spectrum) and $p53_{L344A}$ (lower spectrum) in the presence of DNA-RE p21_I. The DNA-RE is schematically depicted on top of the mass spectra. p53 (circle), p53:DNA-complex (circle with DNA), DnaK (square), unknown 112 kDa species (triangle), unknown 156 kDa species (pentagon), unknown 199 kDa species (star).

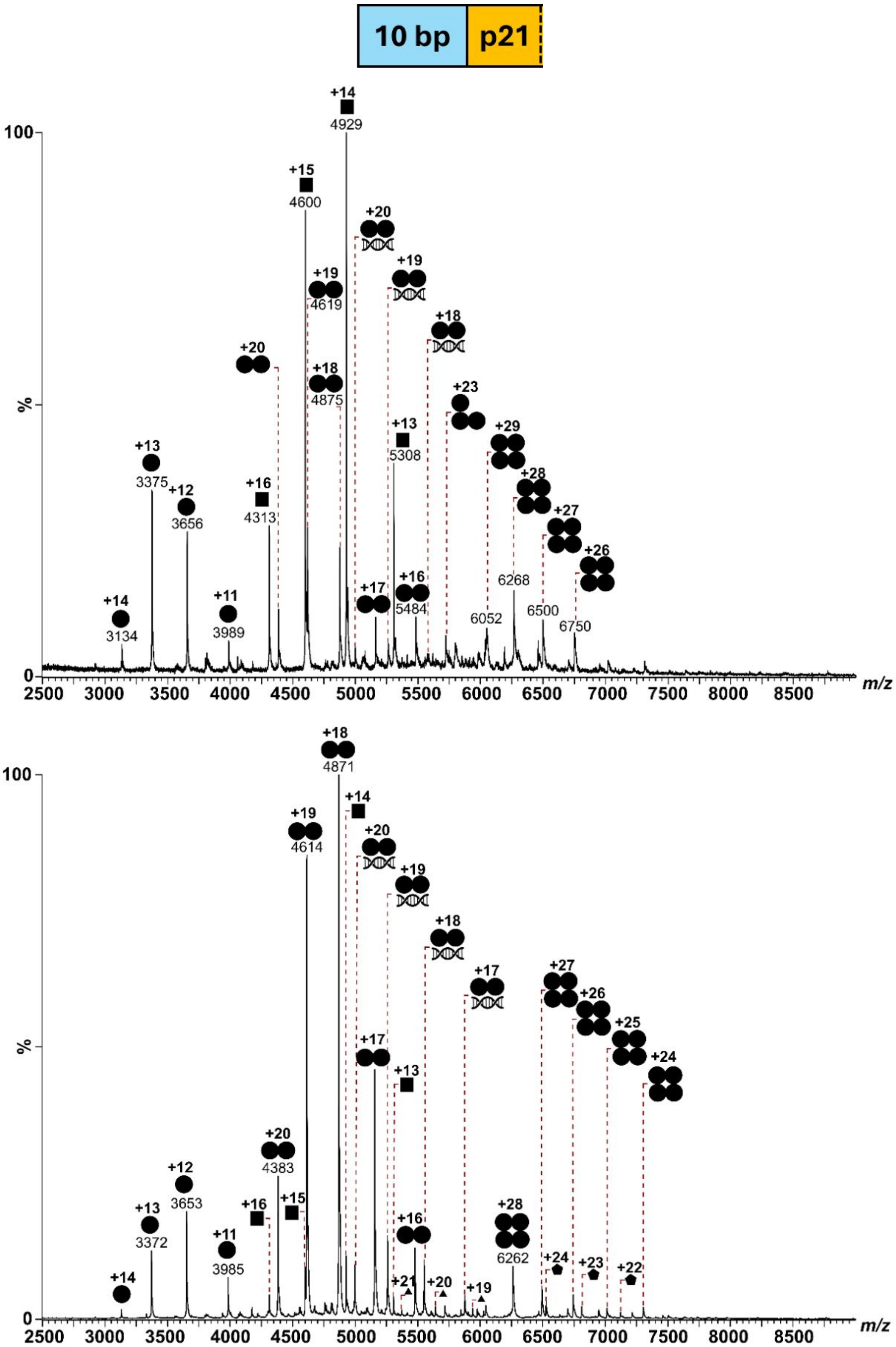

Figure S10. Native mass spectra of $p53_{wild\text{-}type}$ (upper spectrum) and $p53_{L344A}$ (lower spectrum) in the presence of DNA-RE p21_J. The DNA-RE is schematically depicted on top of the mass spectra. p53 (circle), p53:DNA-complex (circle with DNA), DnaK (square), unknown 112 kDa species (triangle), unknown 156 kDa species (pentagon), unknown 199 kDa species (star).

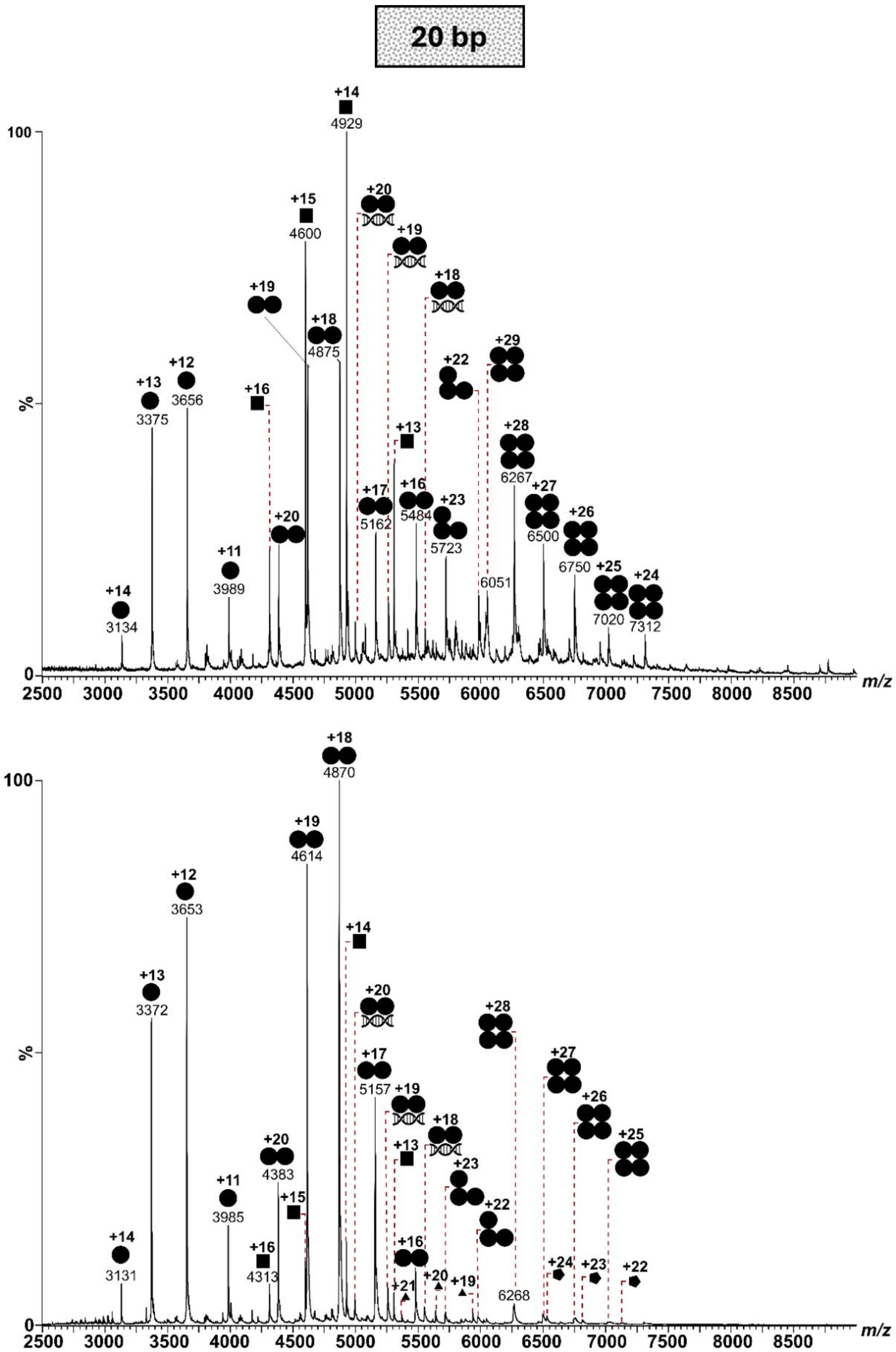

Figure S11. Native mass spectra of $p53_{wild\text{-}type}$ (upper spectrum) and $p53_{L344A}$ (lower spectrum) in the presence of DNA-RE p21_K. The DNA-RE is schematically depicted on top of the mass spectra. p53 (circle), p53:DNA-complex (circle with DNA), DnaK (square), unknown 112 kDa species (triangle), unknown 156 kDa species (pentagon), unknown 199 kDa species (star).

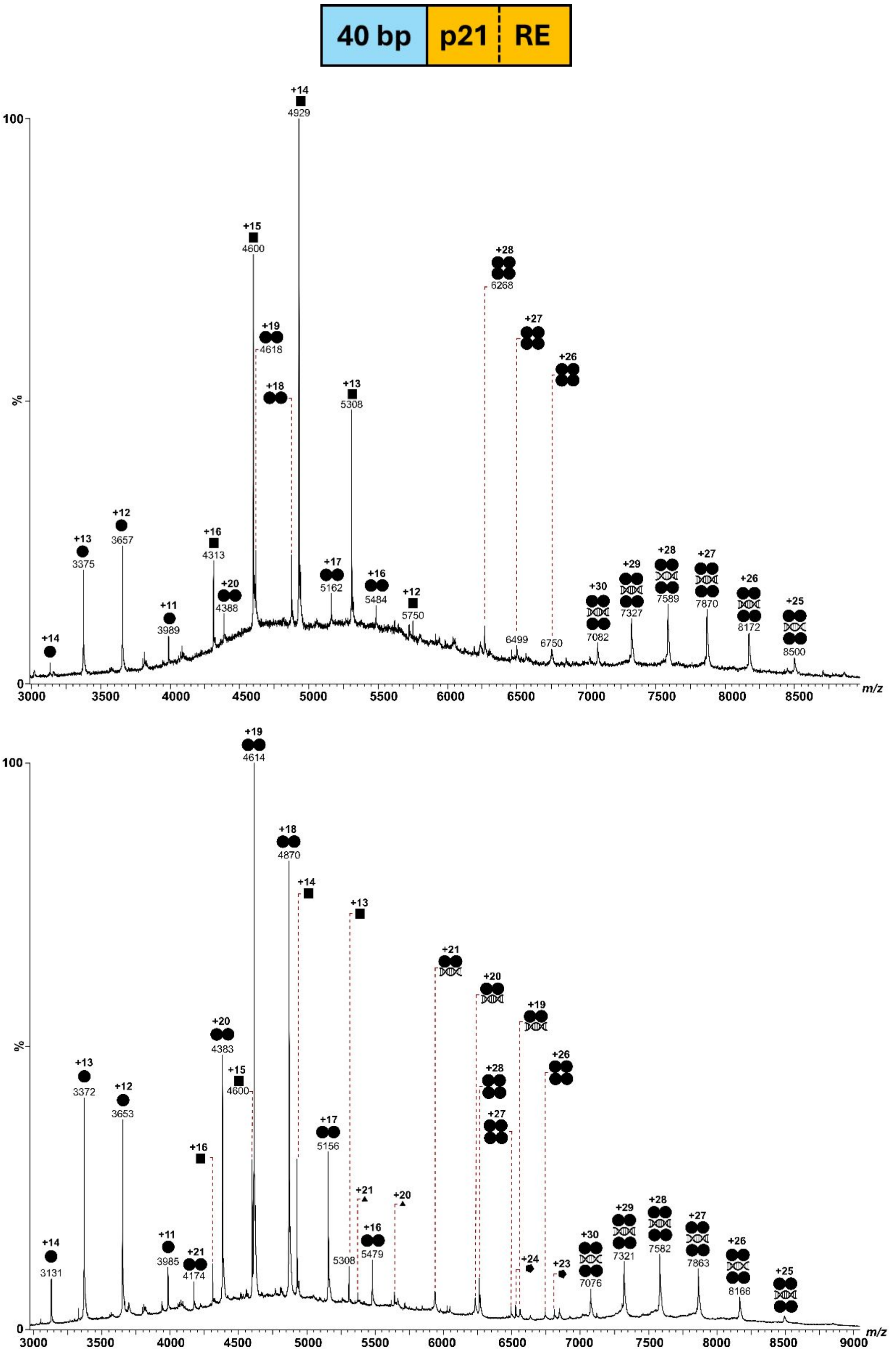

Figure S12. Native mass spectra of $p53_{wild\text{-}type}$ (upper spectrum) and $p53_{L344A}$ (lower spectrum) in the presence of DNA-RE p21_L. The DNA-RE is schematically depicted on top of the mass spectra. p53 (circle), p53:DNA-complex (circle with DNA), DnaK (square), unknown 112 kDa species (triangle), unknown 156 kDa species (pentagon), unknown 199 kDa species (star).

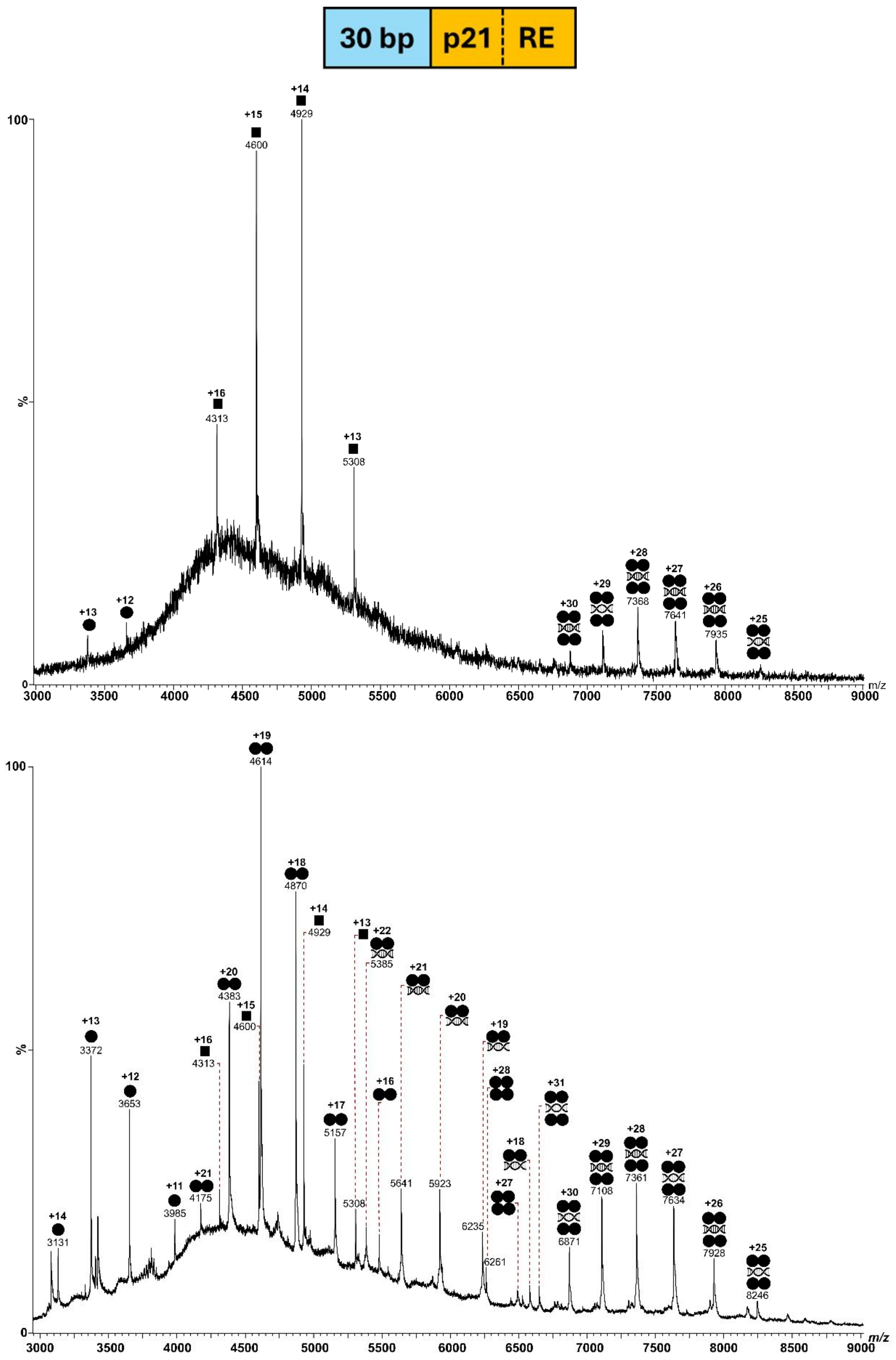

Figure S13. Native mass spectra of $p53_{wild\text{-}type}$ (upper spectrum) and $p53_{L344A}$ (lower spectrum) in the presence of DNA-RE p21_M. The DNA-RE is schematically depicted on top of the mass spectra. p53 (circle), p53:DNA-complex (circle with DNA), DnaK (square), unknown 112 kDa species (triangle), unknown 156 kDa species (pentagon), unknown 199 kDa species (star).

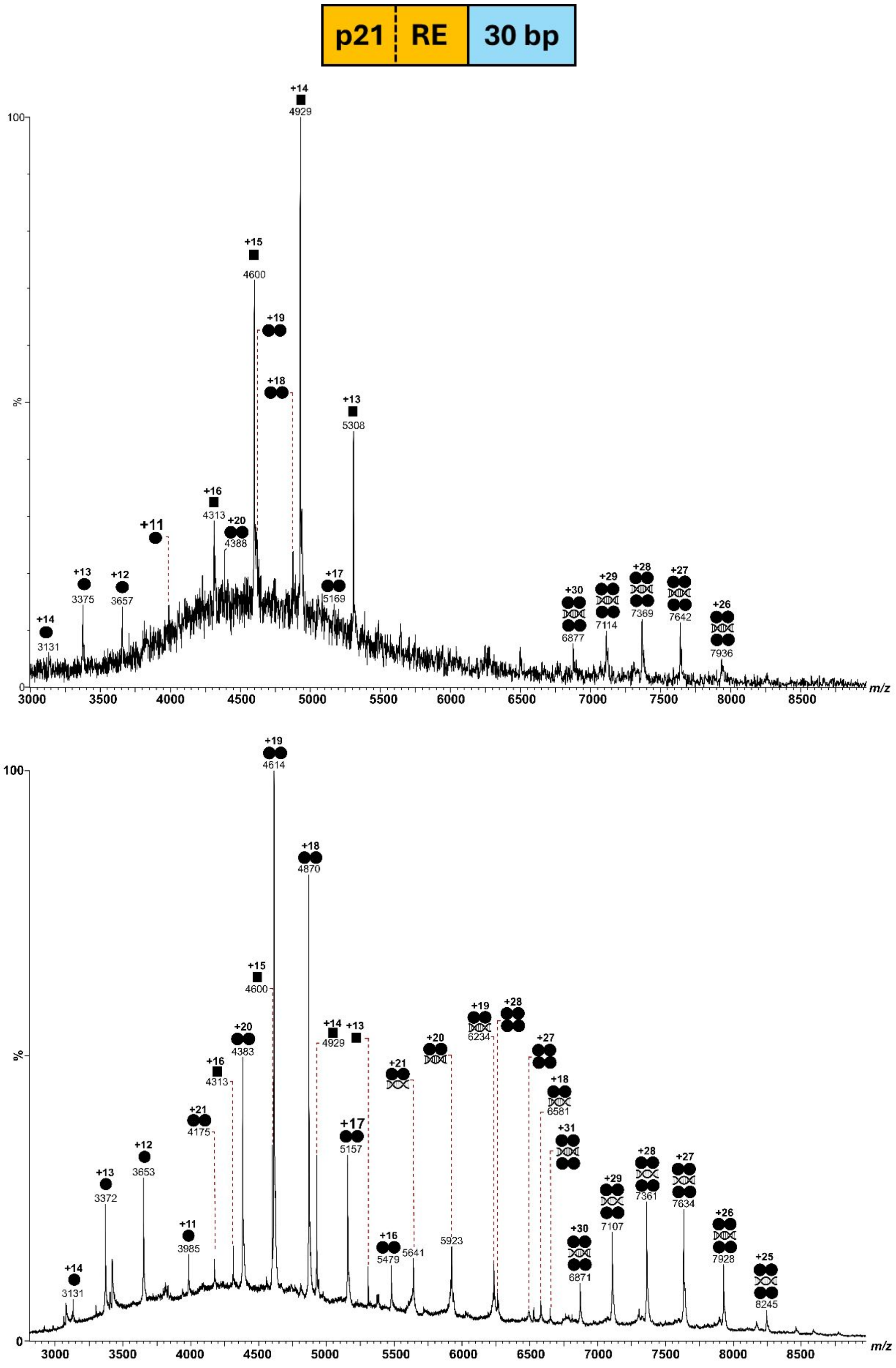

Figure S14. Native mass spectra of p53$_{wild\text{-}type}$ (upper spectrum) and p53$_{L344A}$ (lower spectrum) in the presence of DNA-RE p21_N. The DNA-RE is schematically depicted on top of the mass spectra. p53 (circle), p53:DNA-complex (circle with DNA), DnaK (square), unknown 112 kDa species (triangle), unknown 156 kDa species (pentagon), unknown 199 kDa species (star).

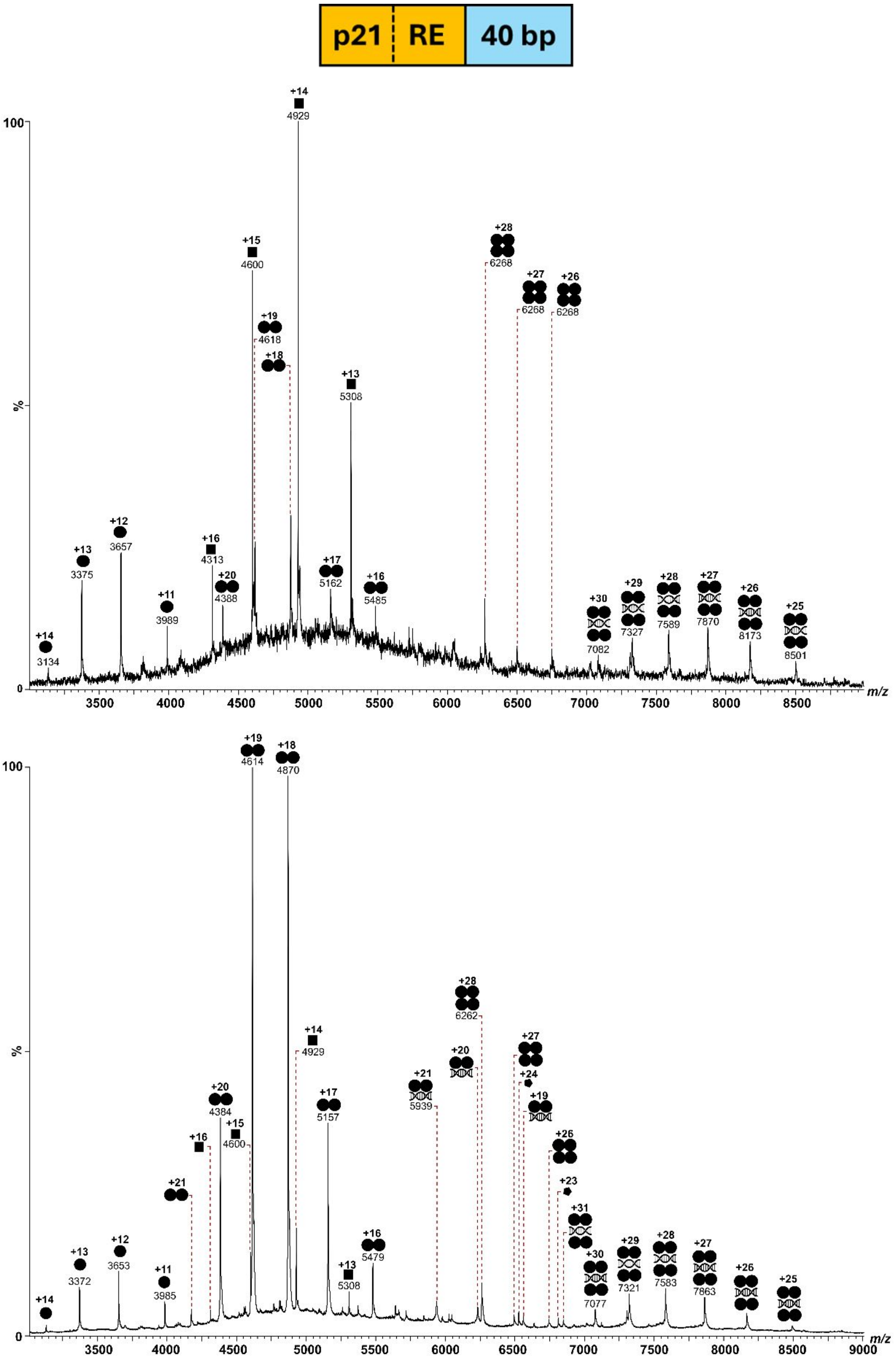

Figure S15. Native mass spectra of $p53_{wild\text{-}type}$ (upper spectrum) and $p53_{L344A}$ (lower spectrum) in the presence of DNA-RE p21_O. The DNA-RE is schematically depicted on top of the mass spectra. p53 (circle), p53:DNA-complex (circle with DNA), DnaK (square), unknown 112 kDa species (triangle), unknown 156 kDa species (pentagon), unknown 199 kDa species (star).

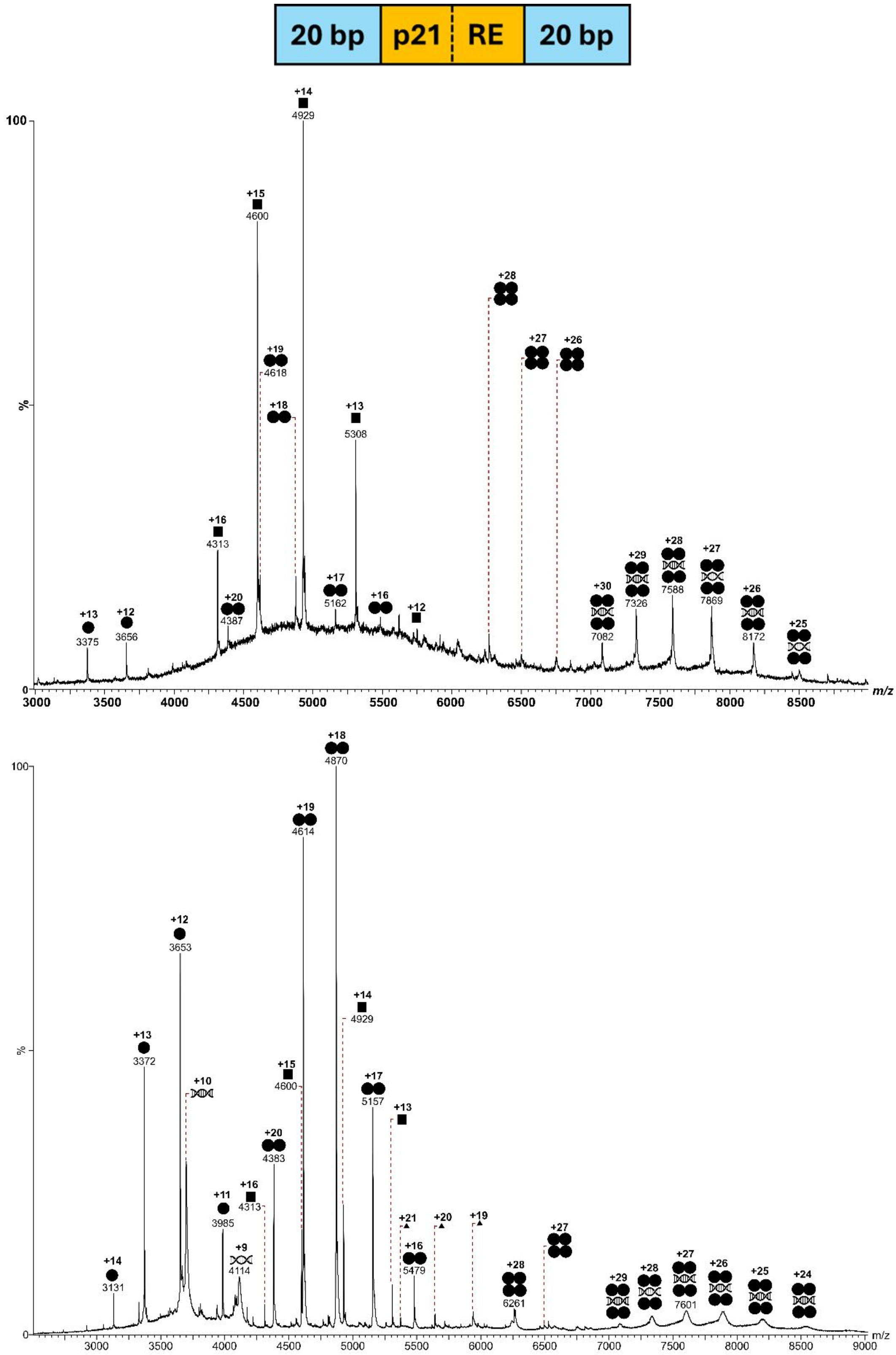

Figure S16. Native mass spectra of p53$_{wild\text{-}type}$ (upper spectrum) and p53$_{L344A}$ (lower spectrum) in the presence of DNA-RE p21_P. The DNA-RE is schematically depicted on top of the mass spectra. p53 (circle), p53:DNA-complex (circle with DNA), DnaK (square), unknown 112 kDa species (triangle), unknown 156 kDa species (pentagon), unknown 199 kDa species (star).

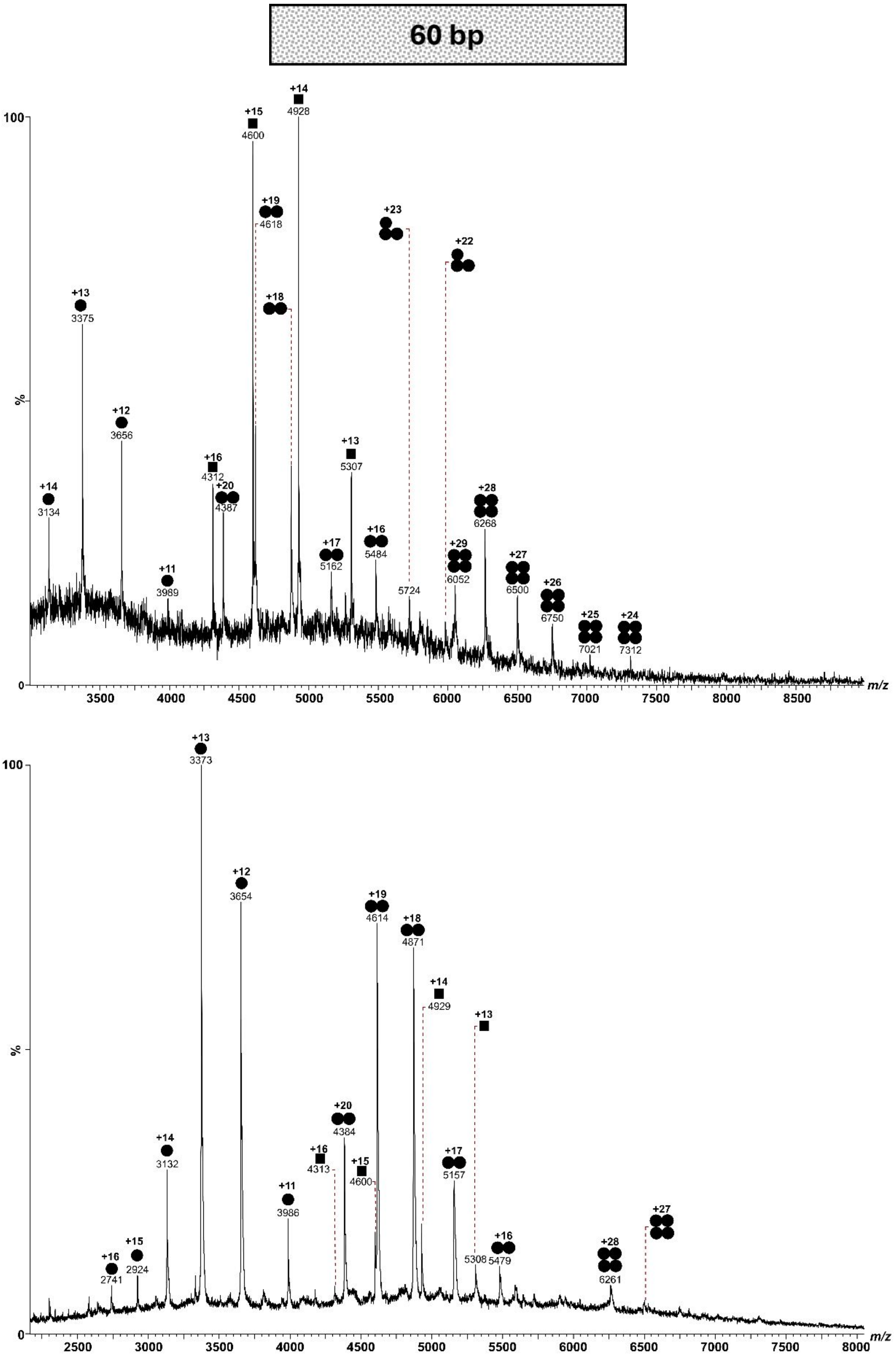

Figure S17. Native mass spectra of $p53_{wild\text{-}type}$ (upper spectrum) and $p53_{L344A}$ (lower spectrum) in the presence of DNA-RE p21_Q. The DNA-RE is schematically depicted on top of the mass spectra. p53 (circle), p53:DNA-complex (circle with DNA), DnaK (square), unknown 112 kDa species (triangle), unknown 156 kDa species (pentagon), unknown 199 kDa species (star).

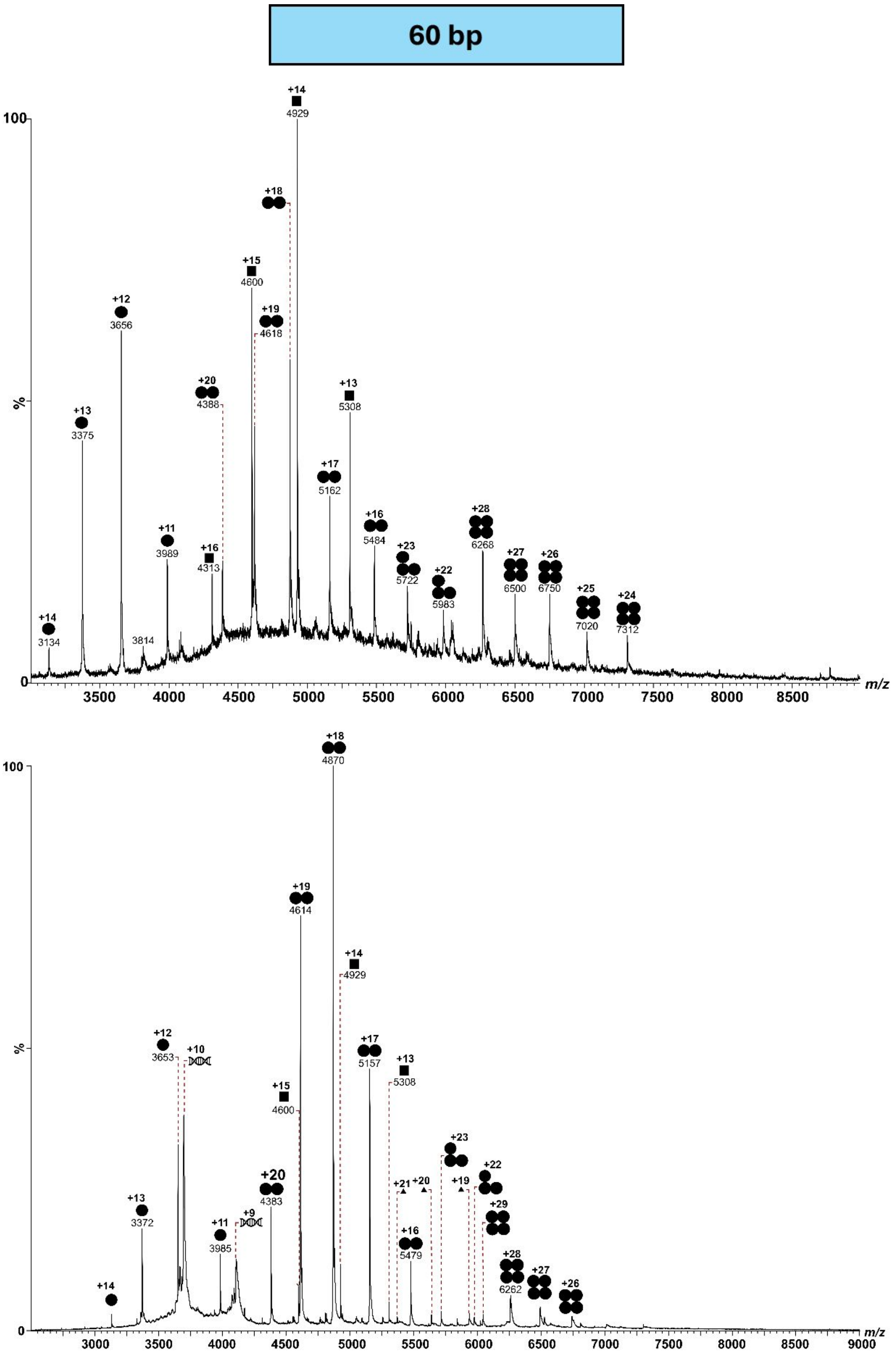

Figure S18. Native mass spectra of $p53_{wild\text{-}type}$ (upper spectrum) and $p53_{L344A}$ (lower spectrum) in the presence of DNA-RE p21_R. The DNA-RE is schematically depicted on top of the mass spectra. p53 (circle), p53:DNA-complex (circle with DNA), DnaK (square), unknown 112 kDa species (triangle), unknown 156 kDa species (pentagon), unknown 199 kDa species (star).

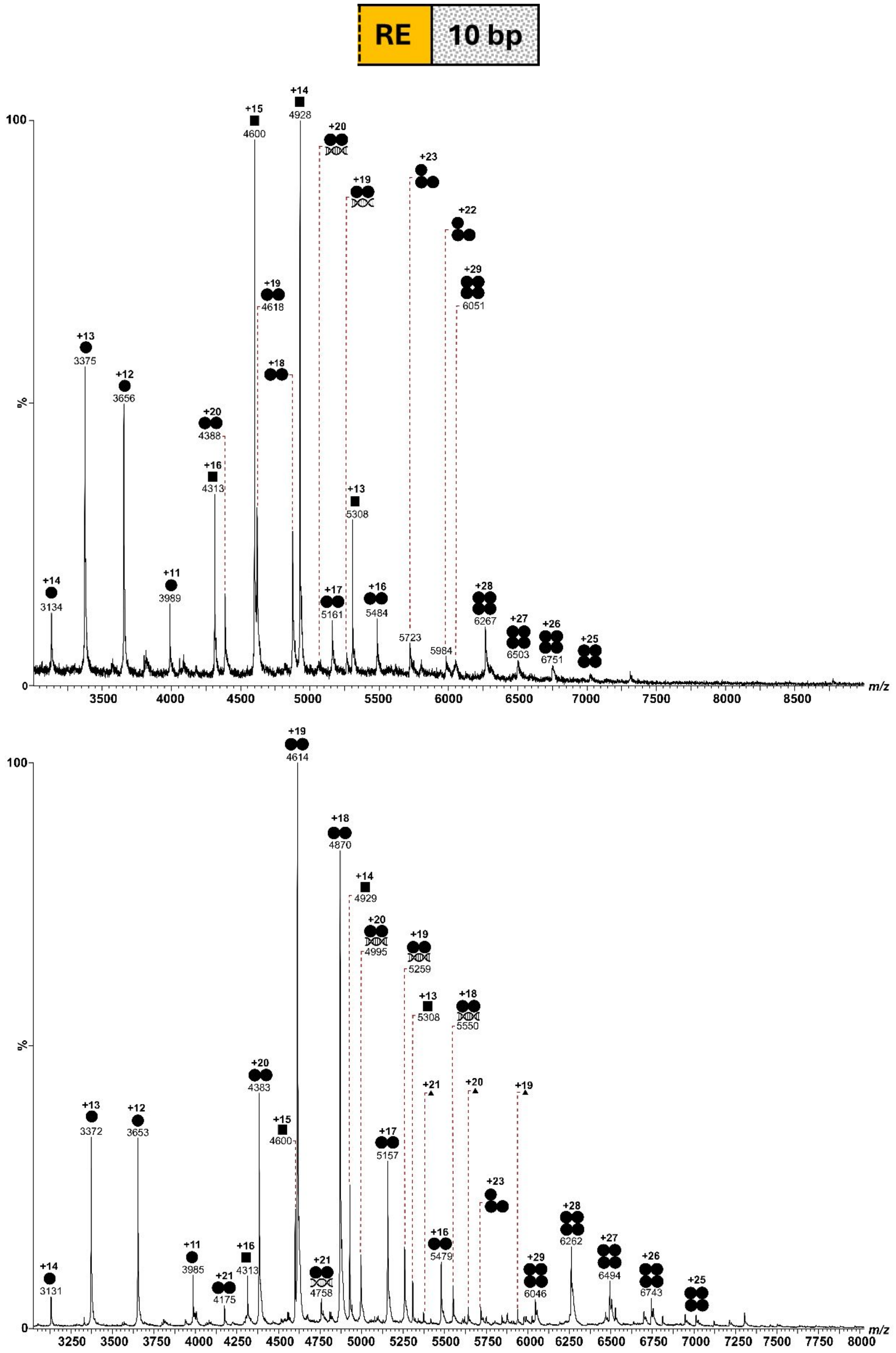

Figure S19. Native mass spectra of $p53_{wild\text{-}type}$ (upper spectrum) and $p53_{L344A}$ (lower spectrum) in the presence of DNA-RE p21_S. The DNA-RE is schematically depicted on top of the mass spectra. p53 (circle), p53:DNA-complex (circle with DNA), DnaK (square), unknown 112 kDa species (triangle), unknown 156 kDa species (pentagon), unknown 199 kDa species (star).

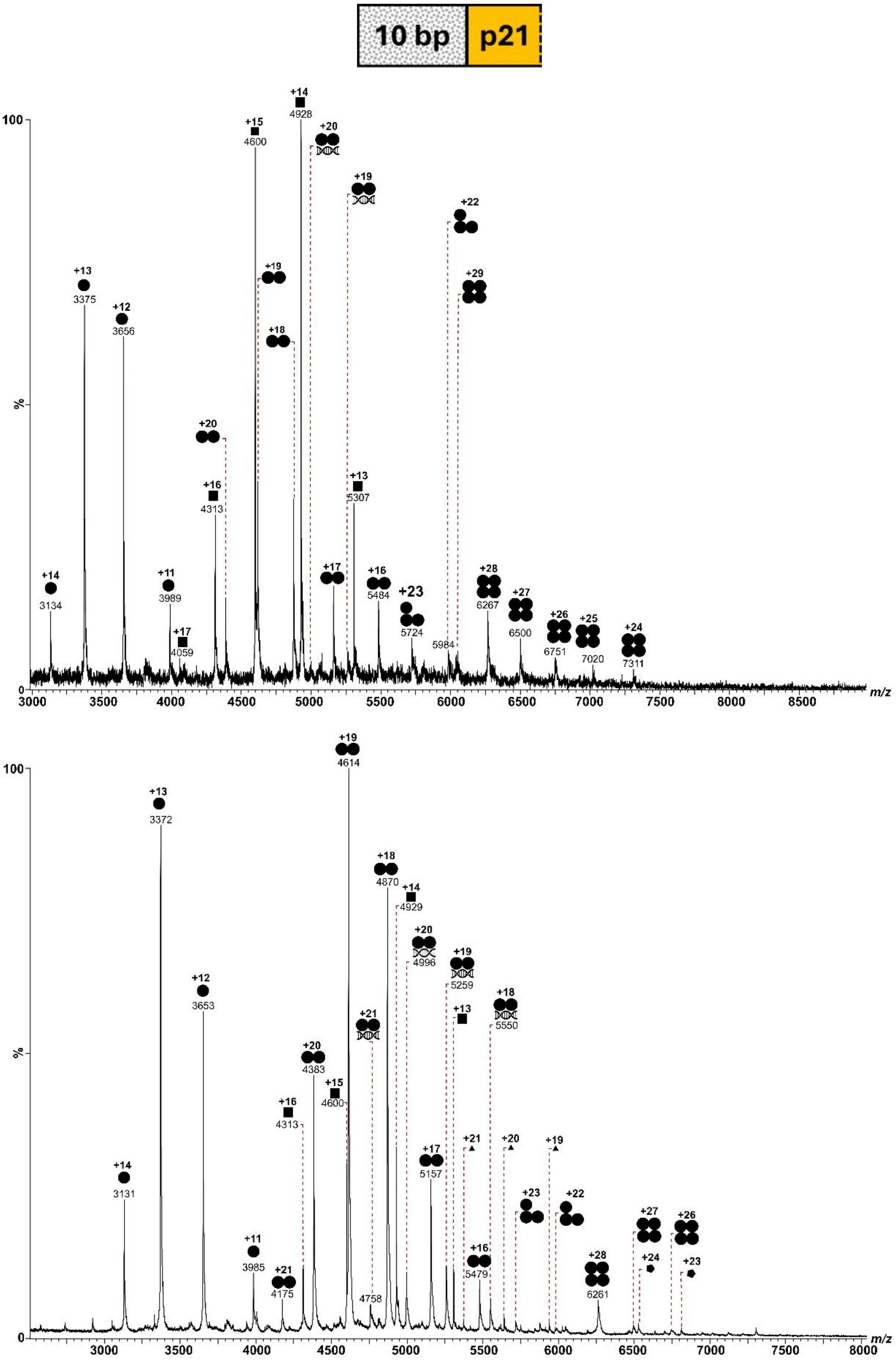

Figure S20. Native mass spectra of $p53_{wild\text{-}type}$ (upper spectrum) and $p53_{L344A}$ (lower spectrum) in the presence of DNA-RE p21_T. The DNA-RE is schematically depicted on top of the mass spectra. p53 (circle), p53:DNA-complex (circle with DNA), DnaK (square), unknown 112 kDa species (triangle), unknown 156 kDa species (pentagon), unknown 199 kDa species (star).

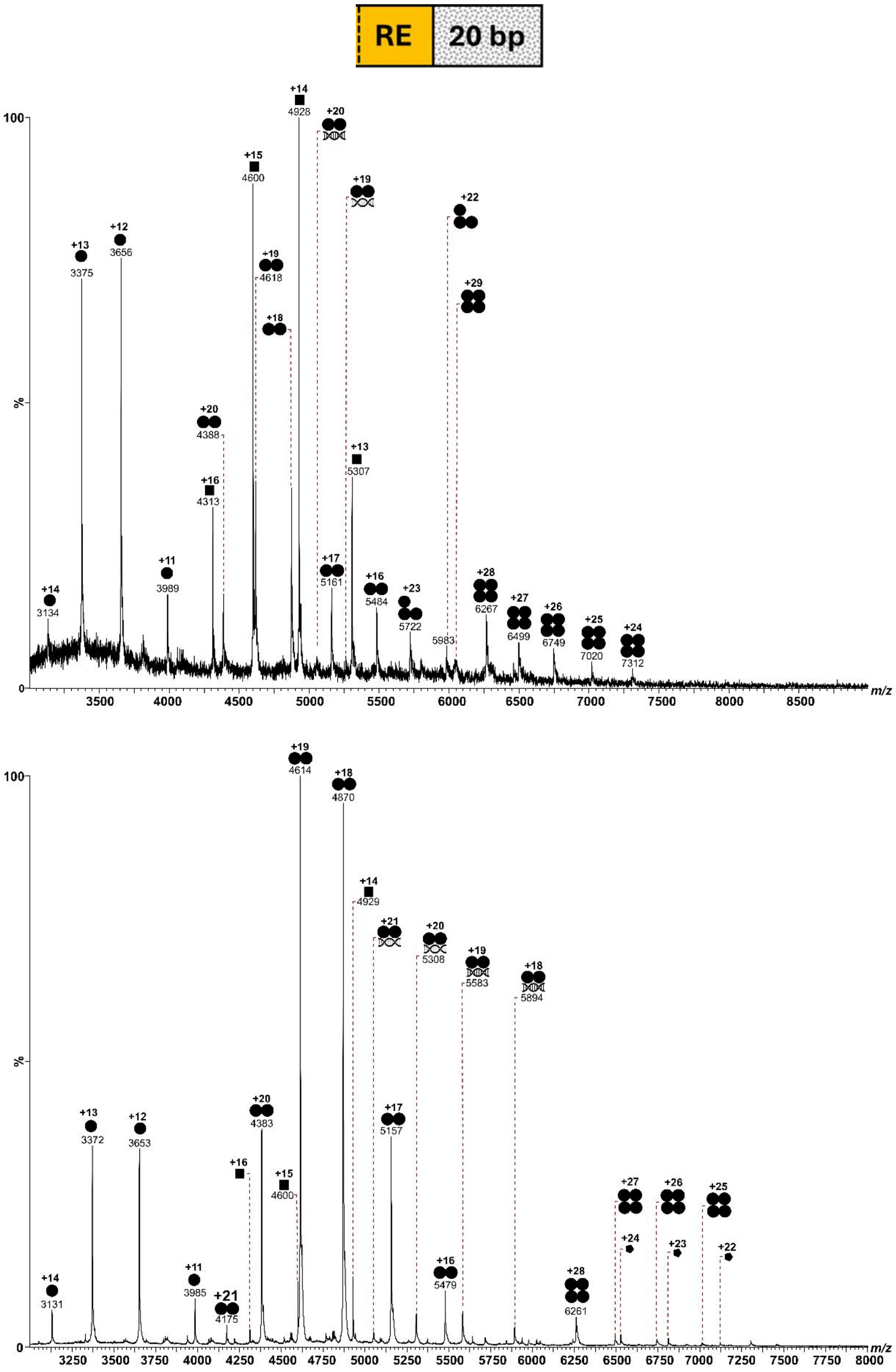

Figure S21. Native mass spectra of $p53_{wild\text{-}type}$ (upper spectrum) and $p53_{L344A}$ (lower spectrum) in the presence of DNA-RE p21_U. The DNA-RE is schematically depicted on top of the mass spectra. p53 (circle), p53:DNA-complex (circle with DNA), DnaK (square), unknown 112 kDa species (triangle), unknown 156 kDa species (pentagon), unknown 199 kDa species (star).

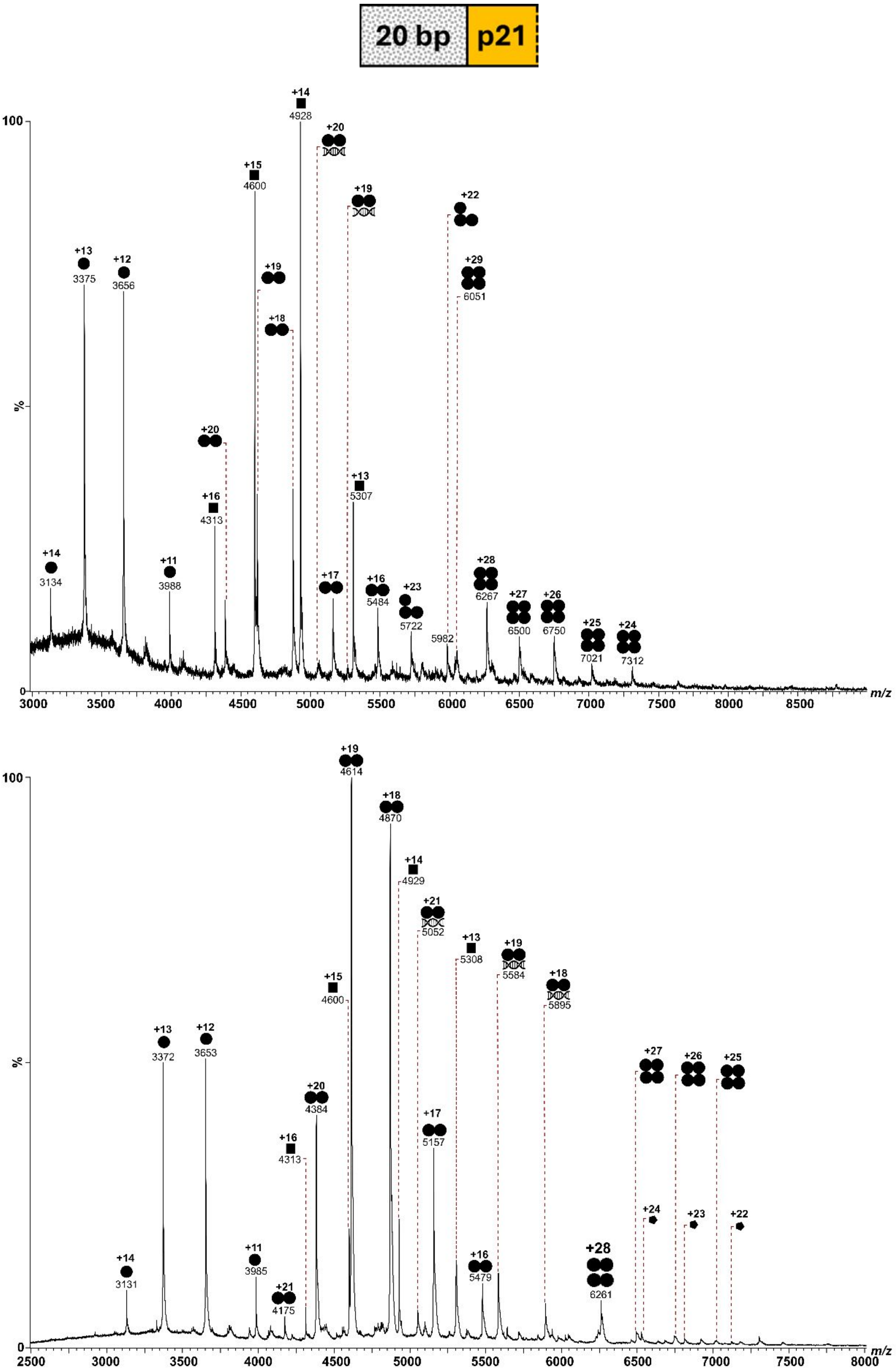

Figure S22. Native mass spectra of p53$_{wild\text{-}type}$ (upper spectrum) and p53$_{L344A}$ (lower spectrum) in the presence of DNA-RE p21_V. The DNA-RE is schematically depicted on top of the mass spectra. p53 (circle), p53:DNA-complex (circle with DNA), DnaK (square), unknown 112 kDa species (triangle), unknown 156 kDa species (pentagon), unknown 199 kDa species (star).

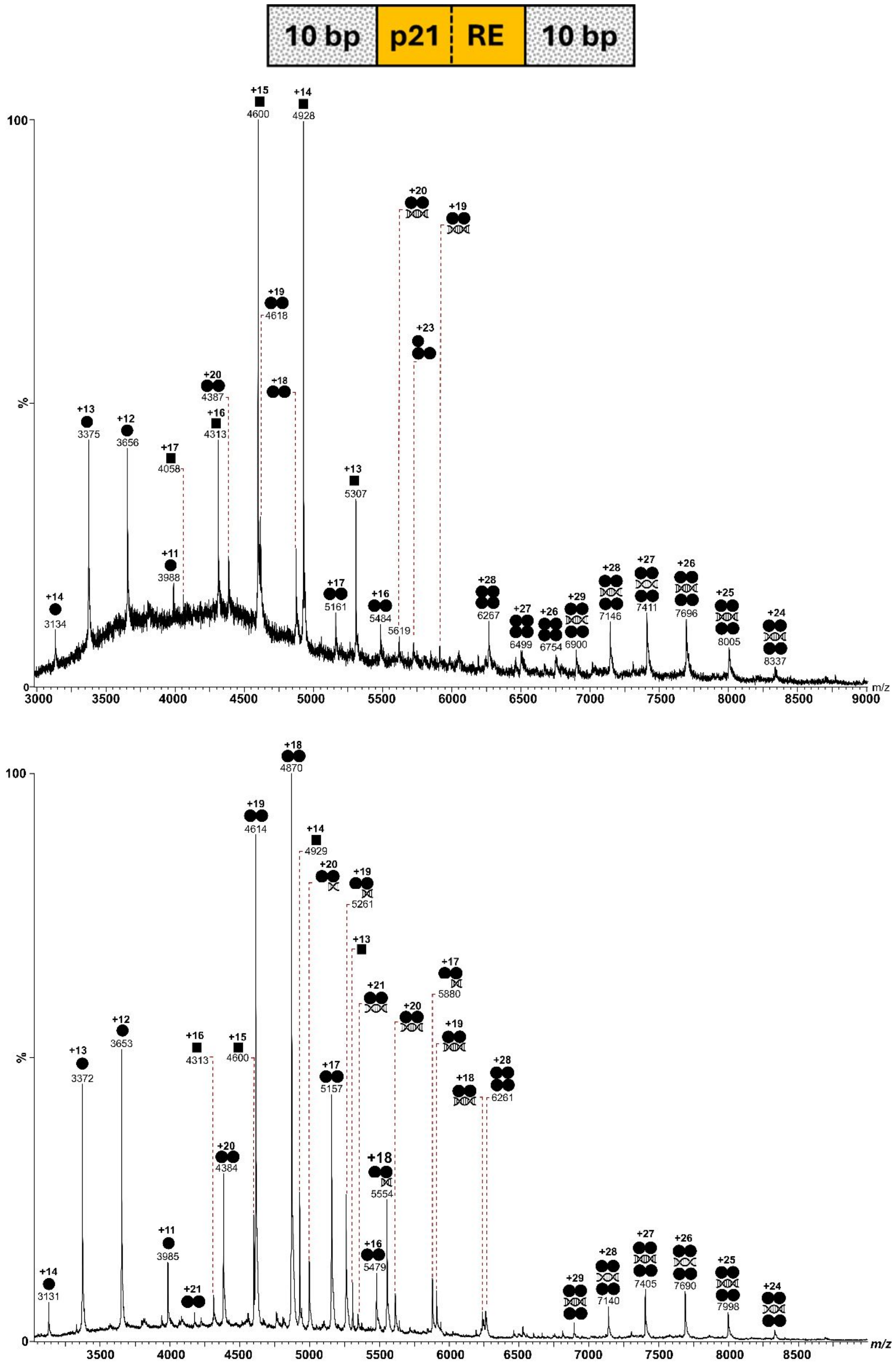

Figure S23. Native mass spectra of $p53_{wild\text{-}type}$ (upper spectrum) and $p53_{L344A}$ (lower spectrum) in the presence of DNA-RE p21_W. The DNA-RE is schematically depicted on top of the mass spectra. p53 (circle), p53:DNA-complex (circle with DNA), DnaK (square), unknown 112 kDa species (triangle), unknown 156 kDa species (pentagon), unknown 199 kDa species (star).

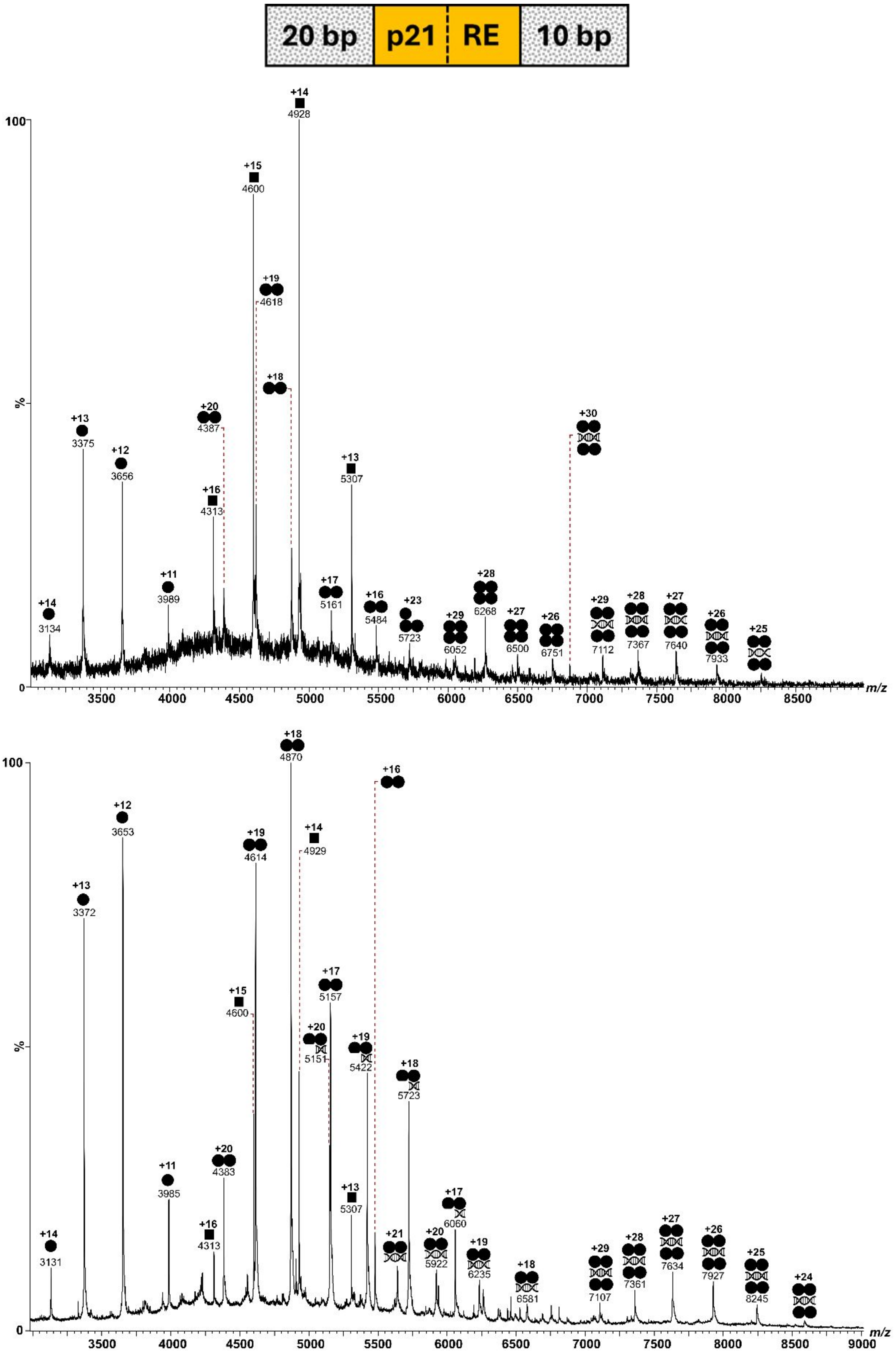

Figure S24. Native mass spectra of p53$_{wild\text{-}type}$ (upper spectrum) and p53$_{L344A}$ (lower spectrum) in the presence of DNA-RE p21_X. The DNA-RE is schematically depicted on top of the mass spectra. p53 (circle), p53:DNA-complex (circle with DNA), DnaK (square), unknown 112 kDa species (triangle), unknown 156 kDa species (pentagon), unknown 199 kDa species (star).

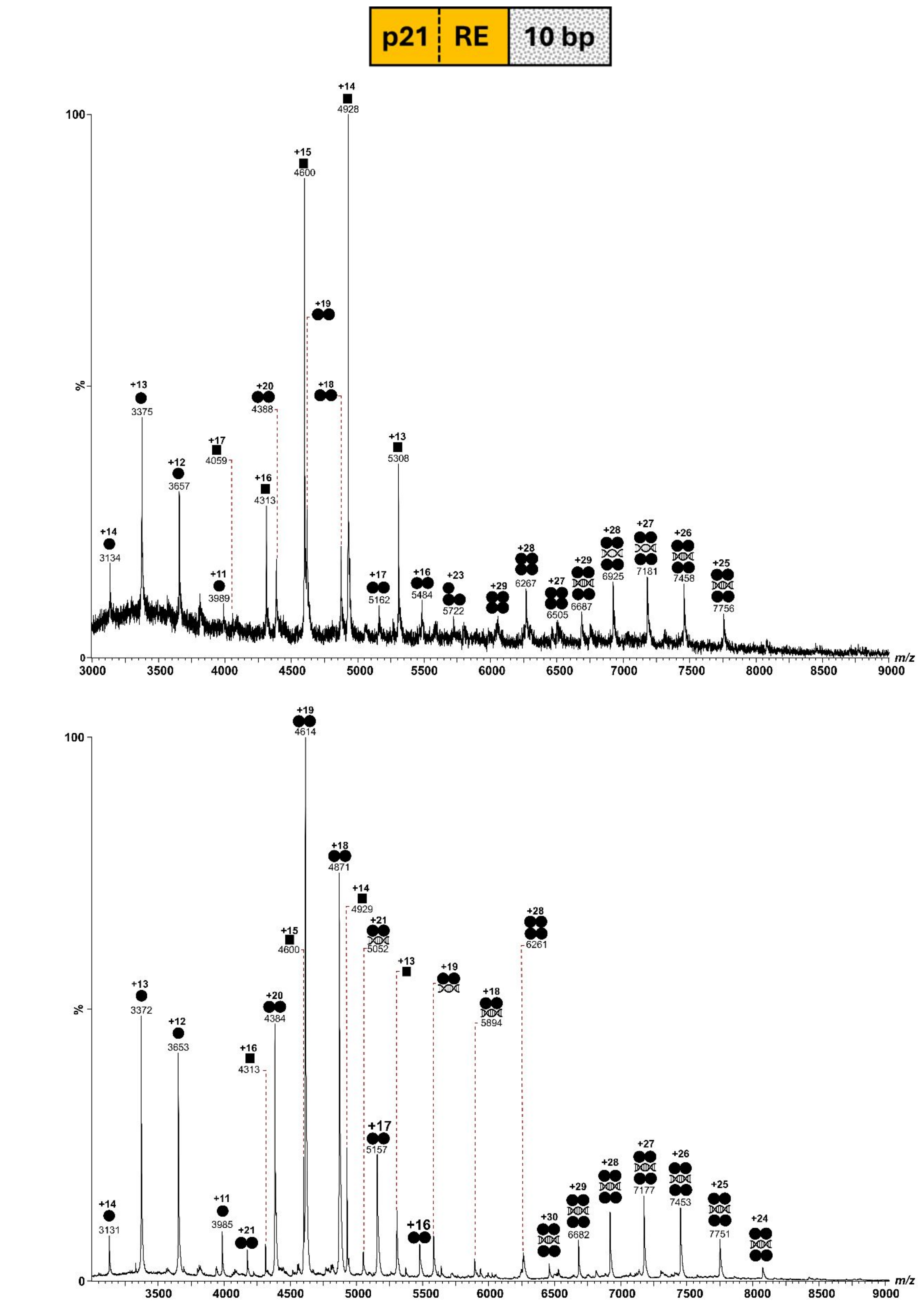

Figure S25. Native mass spectra of p53$_{wild\text{-}type}$ (upper spectrum) and p53$_{L344A}$ (lower spectrum) in the presence of DNA-RE p21_Y. The DNA-RE is schematically depicted on top of the mass spectra. p53 (circle), p53:DNA-complex (circle with DNA), DnaK (square), unknown 112 kDa species (triangle), unknown 156 kDa species (pentagon), unknown 199 kDa species (star).

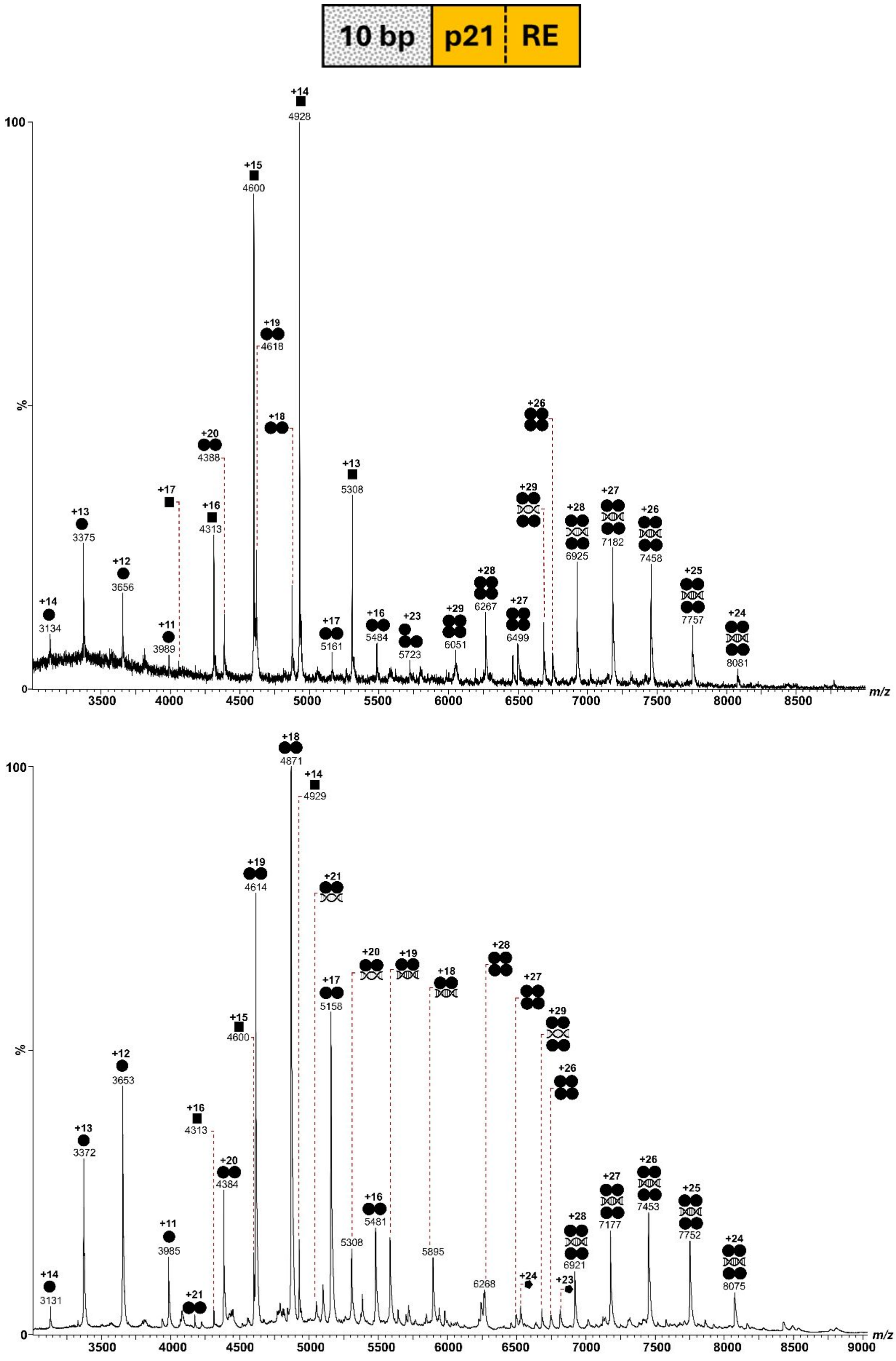

Figure S26. Native mass spectra of $p53_{wild\text{-}type}$ (upper spectrum) and $p53_{L344A}$ (lower spectrum) in the presence of DNA-RE p21_Z. The DNA-RE is schematically depicted on top of the mass spectra. p53 (circle), p53:DNA-complex (circle with DNA), DnaK (square), unknown 112 kDa species (triangle), unknown 156 kDa species (pentagon), unknown 199 kDa species (star).

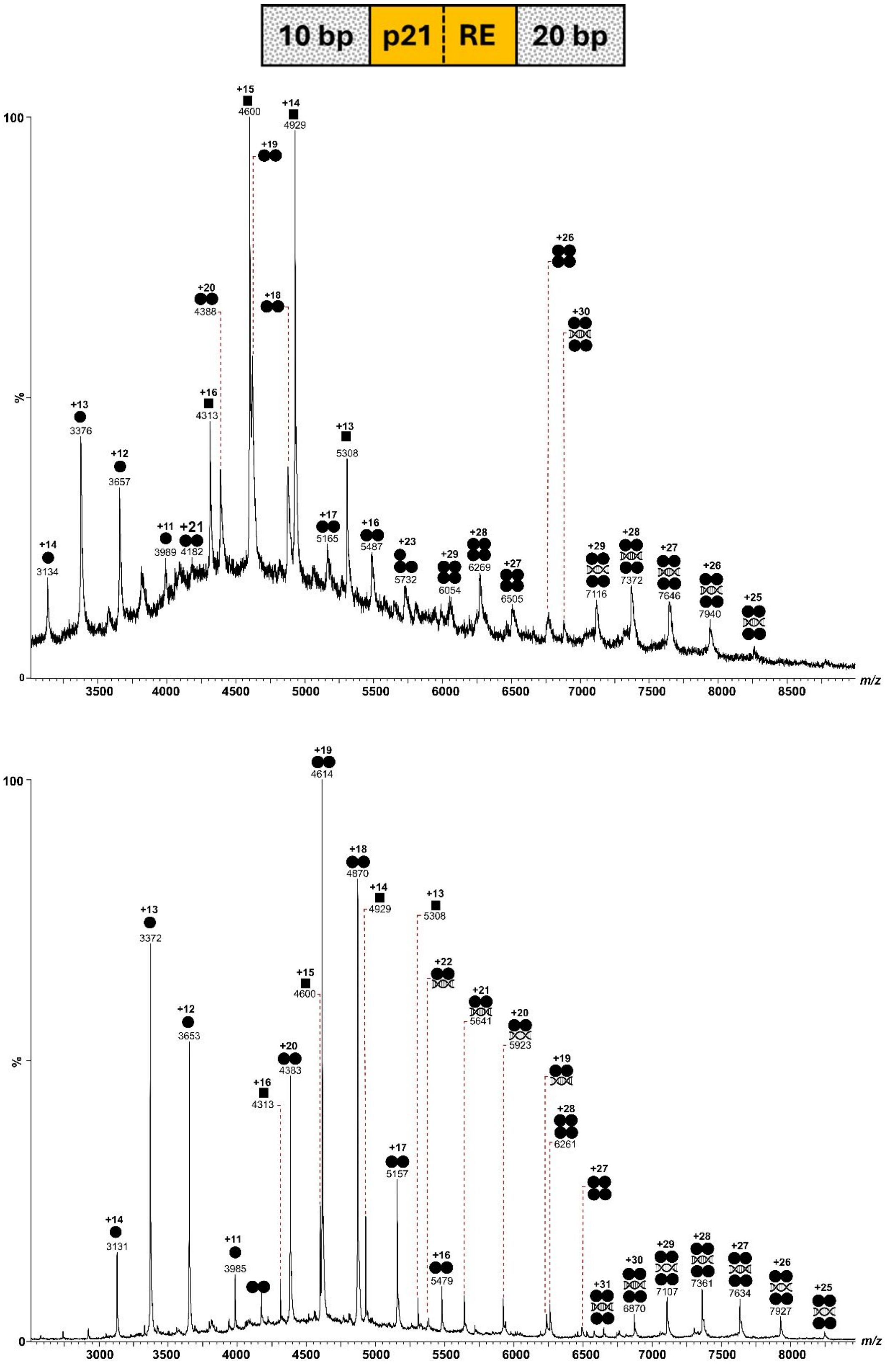

Figure S27. Native mass spectra of p53$_{wild\text{-}type}$ (upper spectrum) and p53$_{L344A}$ (lower spectrum) in the presence of DNA-RE p21_1. The DNA-RE is schematically depicted on top of the mass spectra. p53 (circle), p53:DNA-complex (circle with DNA), DnaK (square), unknown 112 kDa species (triangle), unknown 156 kDa species (pentagon), unknown 199 kDa species (star).

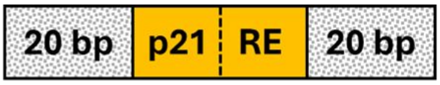

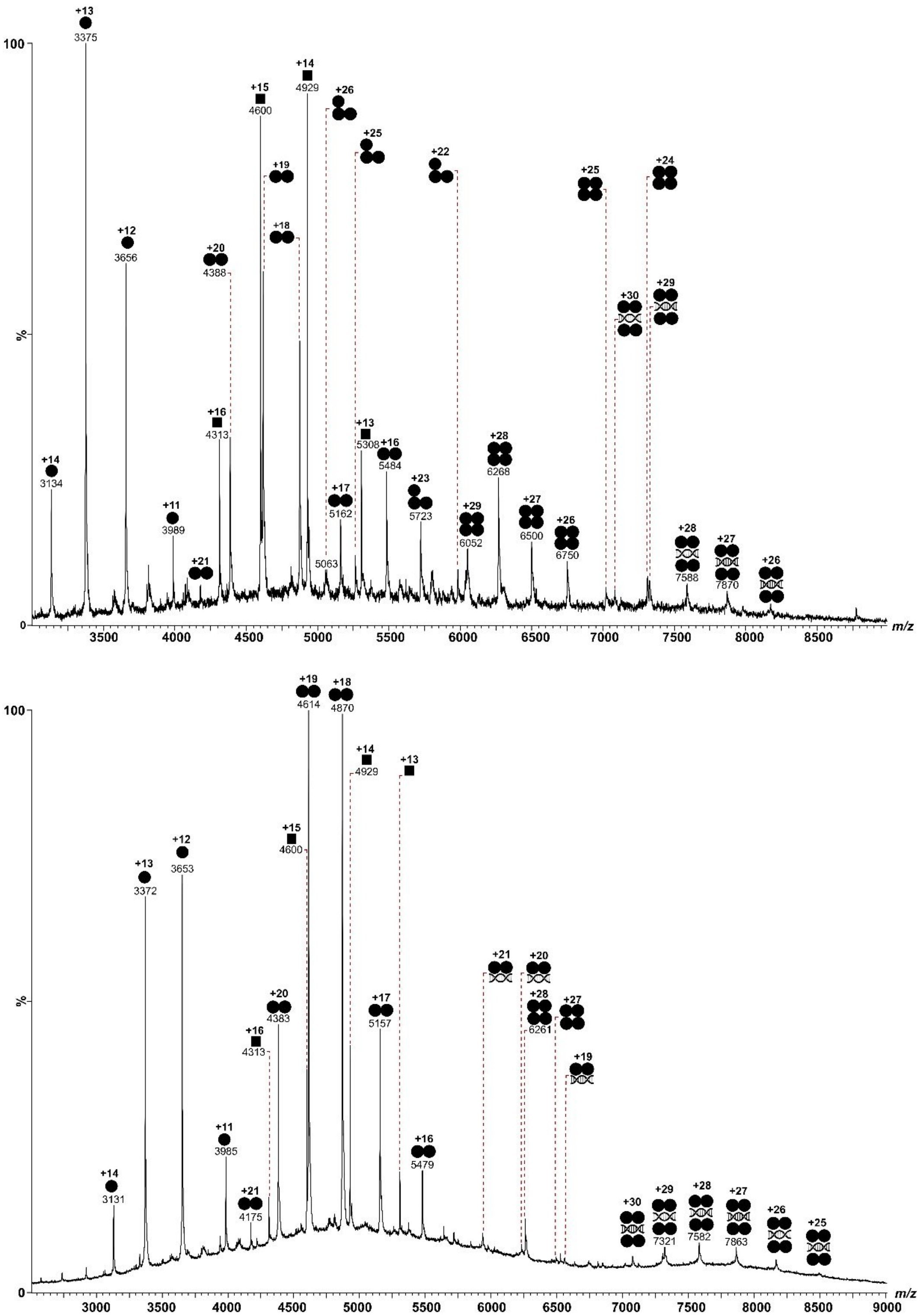

Figure S28. Native mass spectra of p53$_{wild\text{-}type}$ (upper spectrum) and p53$_{L344A}$ (lower spectrum) in the presence of DNA-RE p21_2. The DNA-RE is schematically depicted on top of the mass spectra. p53 (circle), p53:DNA-complex (circle with DNA), DnaK (square), unknown 112 kDa species (triangle), unknown 156 kDa species (pentagon), unknown 199 kDa species (star).

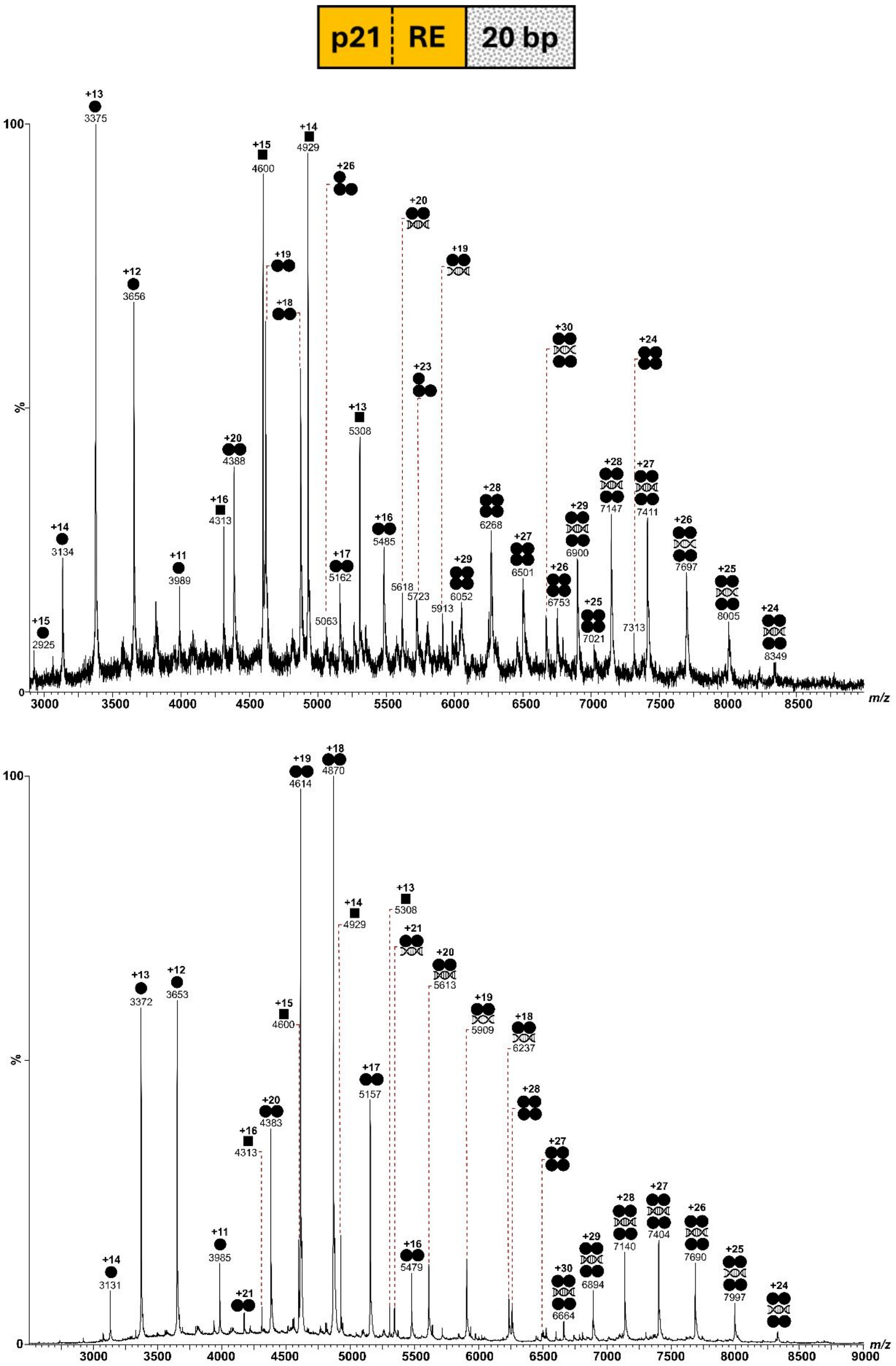

Figure S29. Native mass spectra of $p53_{wild\text{-}type}$ (upper spectrum) and $p53_{L344A}$ (lower spectrum) in the presence of DNA-RE p21_3. The DNA-RE is schematically depicted on top of the mass spectra. p53 (circle), p53:DNA-complex (circle with DNA), DnaK (square), unknown 112 kDa species (triangle), unknown 156 kDa species (pentagon), unknown 199 kDa species (star).

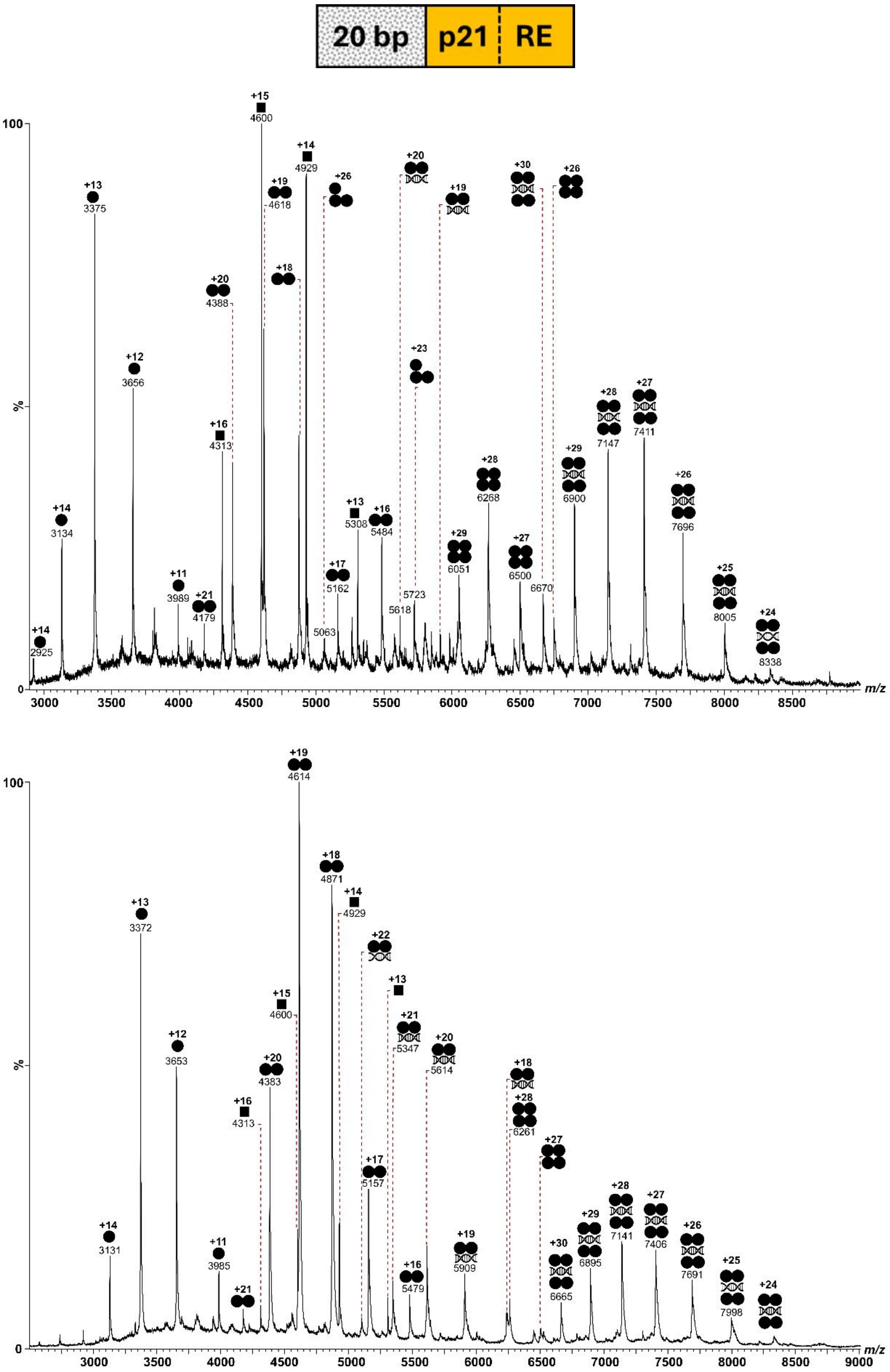

Figure S30. Native mass spectra of $p53_{wild\text{-}type}$ (upper spectrum) and $p53_{L344A}$ (lower spectrum) in the presence of DNA-RE p21_4. The DNA-RE is schematically depicted on top of the mass spectra. p53 (circle), p53:DNA-complex (circle with DNA), DnaK (square), unknown 112 kDa species (triangle), unknown 156 kDa species (pentagon), unknown 199 kDa species (star).

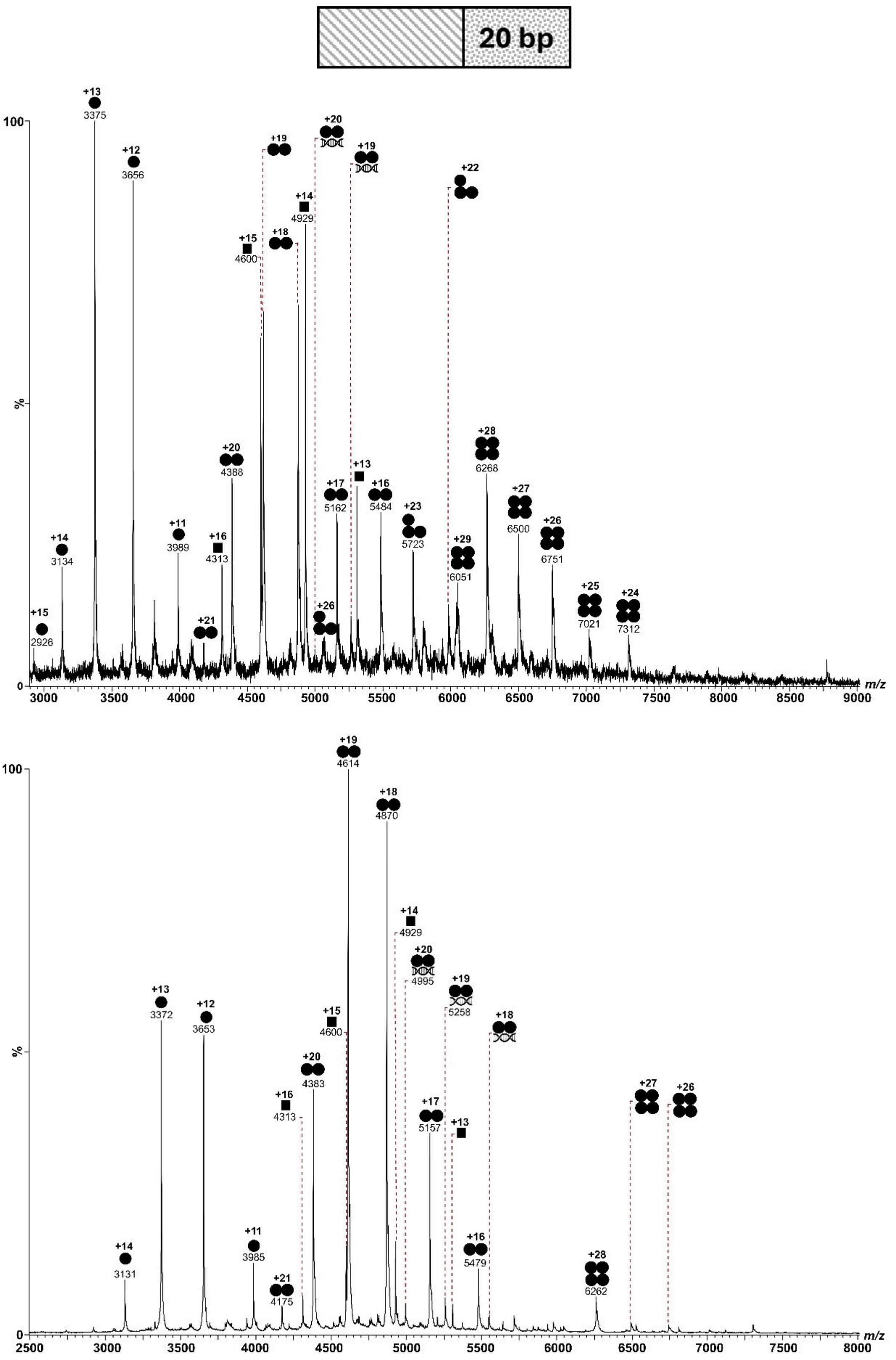

Figure S31. Native mass spectra of $p53_{wild\text{-}type}$ (upper spectrum) and $p53_{L344A}$ (lower spectrum) in the presence of DNA-RE p21_5. The DNA-RE is schematically depicted on top of the mass spectra. p53 (circle), p53:DNA-complex (circle with DNA), DnaK (square), unknown 112 kDa species (triangle), unknown 156 kDa species (pentagon), unknown 199 kDa species (star).

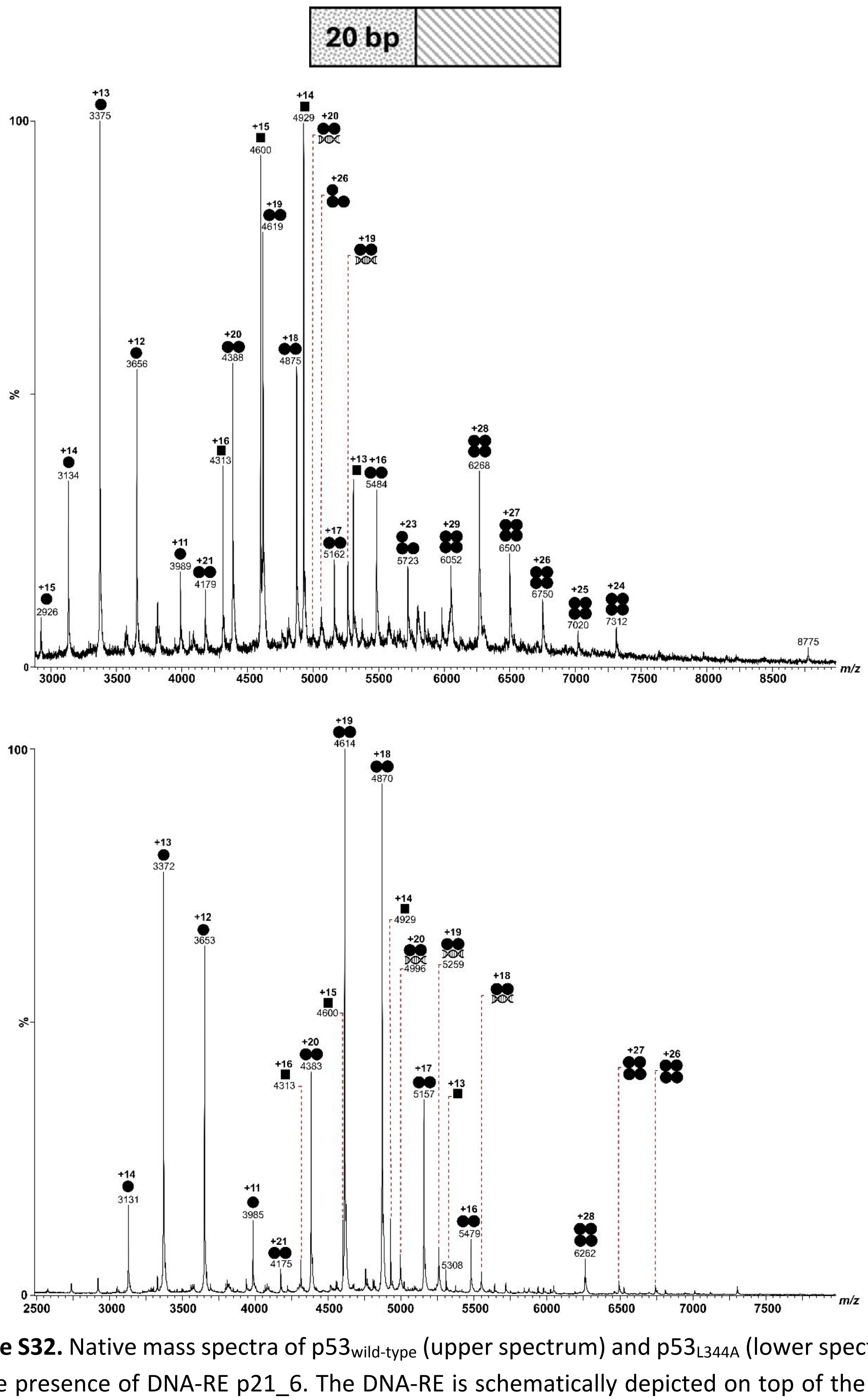

Figure S32. Native mass spectra of $p53_{wild\text{-}type}$ (upper spectrum) and $p53_{L344A}$ (lower spectrum) in the presence of DNA-RE p21_6. The DNA-RE is schematically depicted on top of the mass spectra. p53 (circle), p53:DNA-complex (circle with DNA), DnaK (square), unknown 112 kDa species (triangle), unknown 156 kDa species (pentagon), unknown 199 kDa species (star).

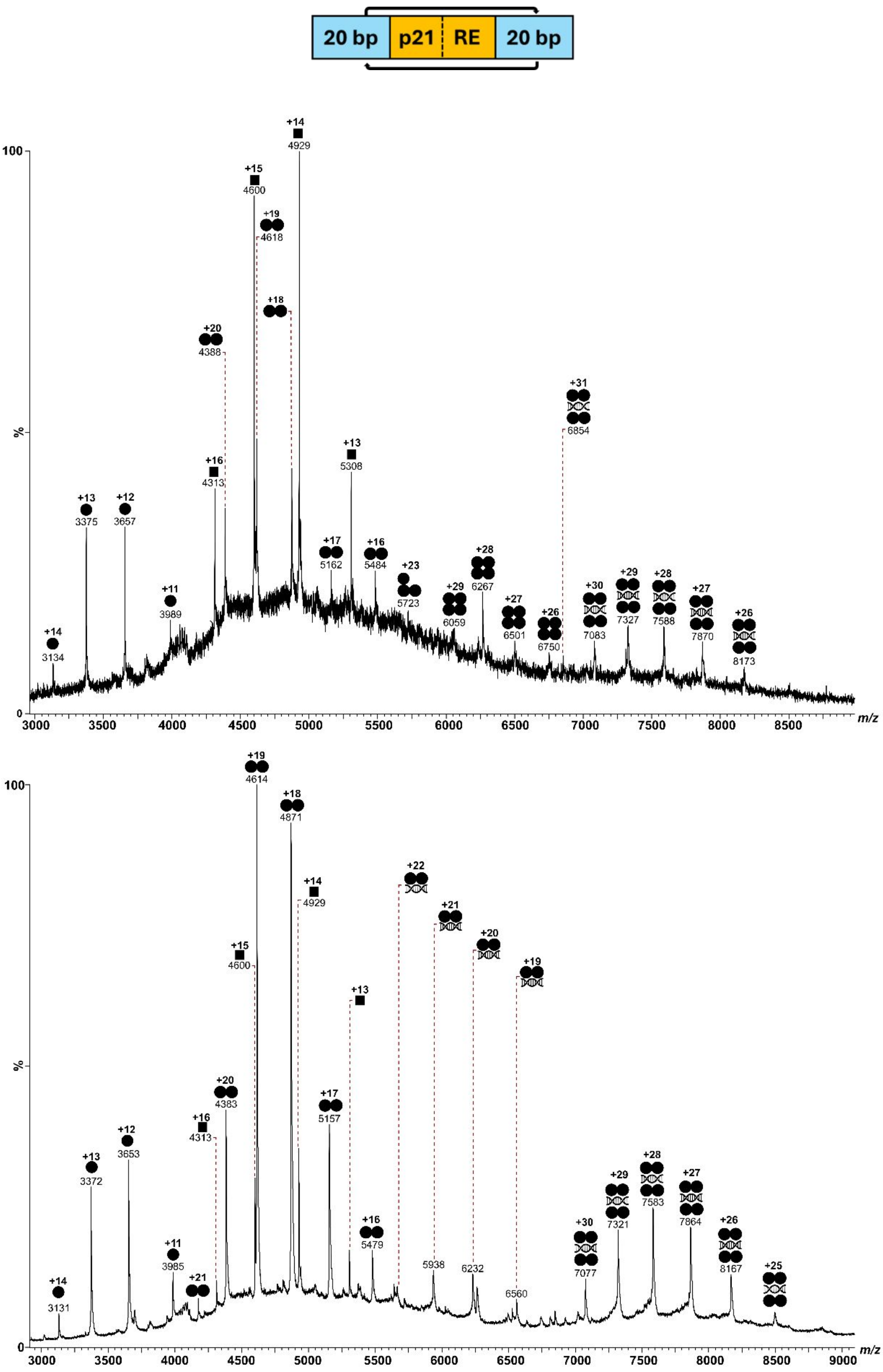

Figure S33. Native mass spectra of $p53_{wild\text{-}type}$ (upper spectrum) and $p53_{L344A}$ (lower spectrum) in the presence of DNA-RE p21_7. The DNA-RE is schematically depicted on top of the mass spectra. p53 (circle), p53:DNA-complex (circle with DNA), DnaK (square), unknown 112 kDa species (triangle), unknown 156 kDa species (pentagon), unknown 199 kDa species (star).

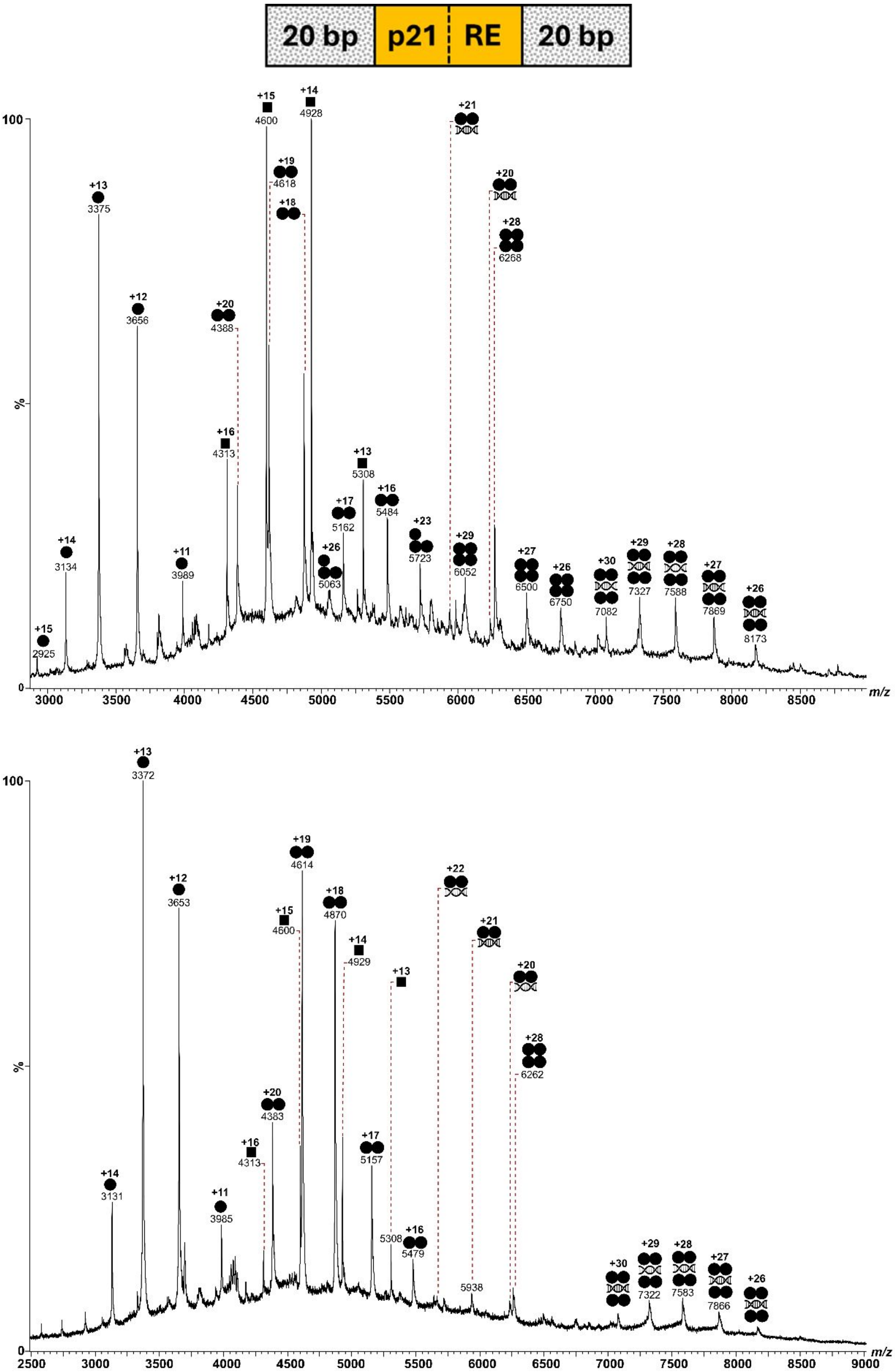

Figure S34. Native mass spectra of $p53_{wild\text{-}type}$ (upper spectrum) and $p53_{L344A}$ (lower spectrum) in the presence of DNA-RE p21_8. The DNA-RE is schematically depicted on top of the mass spectra. p53 (circle), p53:DNA-complex (circle with DNA), DnaK (square), unknown 112 kDa species (triangle), unknown 156 kDa species (pentagon), unknown 199 kDa species (star).

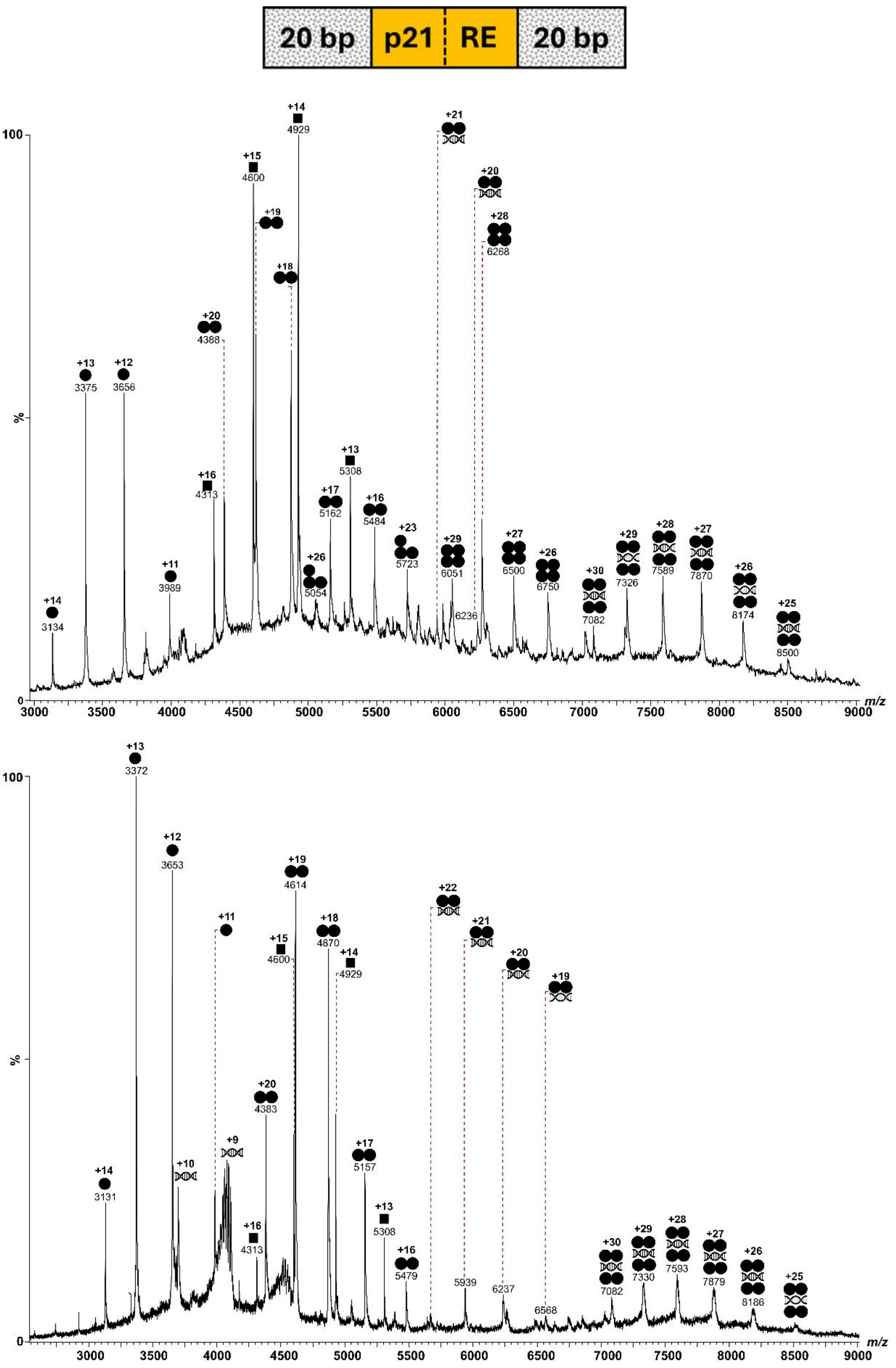

Figure S35. Native mass spectra of $p53_{wild\text{-}type}$ (upper spectrum) and $p53_{L344A}$ (lower spectrum) in the presence of DNA-RE p21_9. The DNA-RE is schematically depicted on top of the mass spectra. p53 (circle), p53:DNA-complex (circle with DNA), DnaK (square), unknown 112 kDa species (triangle), unknown 156 kDa species (pentagon), unknown 199 kDa species (star).

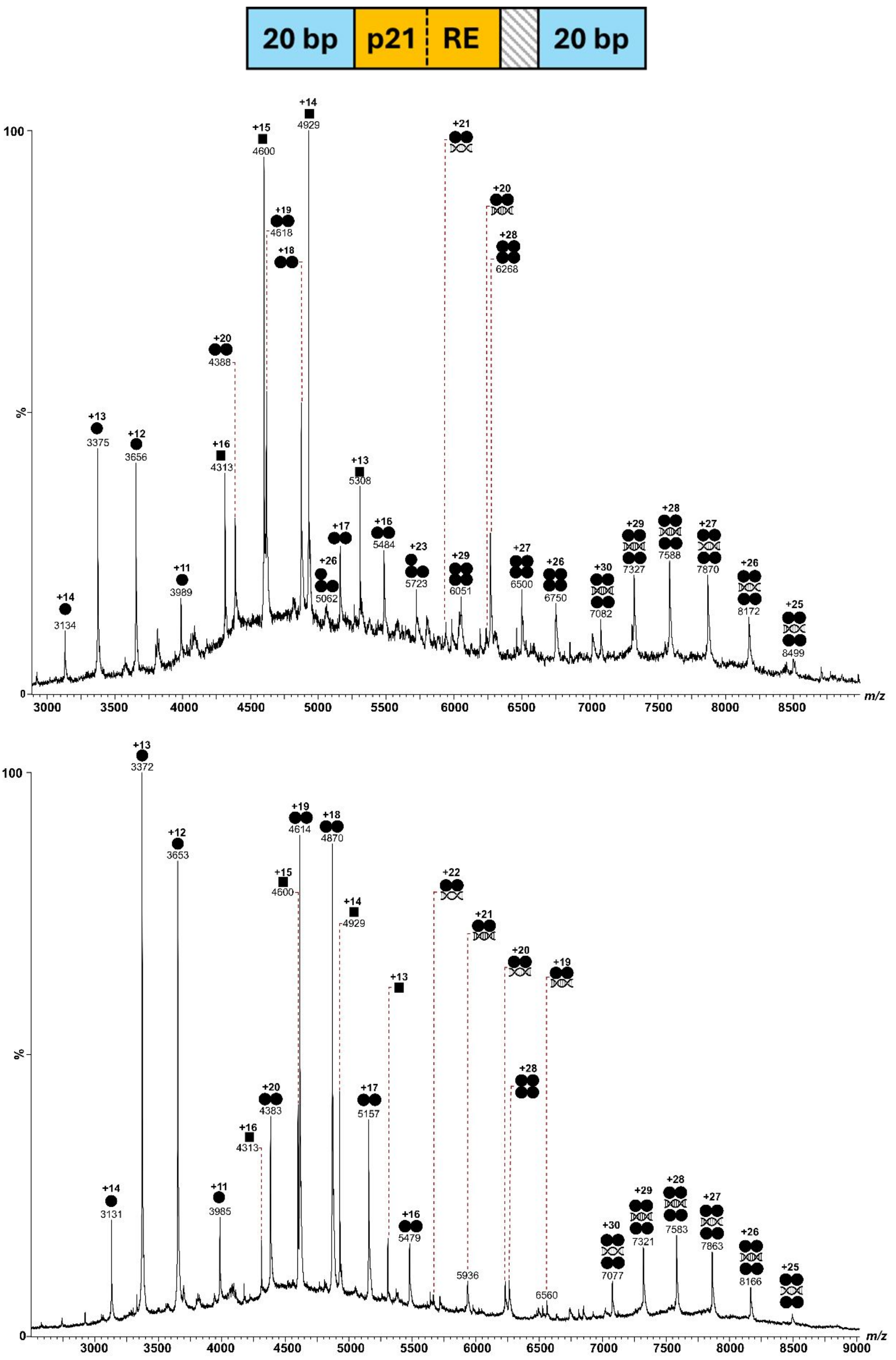

Figure S36. Native mass spectra of $p53_{wild\text{-}type}$ (upper spectrum) and $p53_{L344A}$ (lower spectrum) in the presence of DNA-RE p21_10. The DNA-RE is schematically depicted on top of the mass spectra.p53 (circle), p53:DNA-complex (circle with DNA), DnaK (square), unknown 112 kDa species (triangle), unknown 156 kDa species (pentagon), unknown 199 kDa species (star).

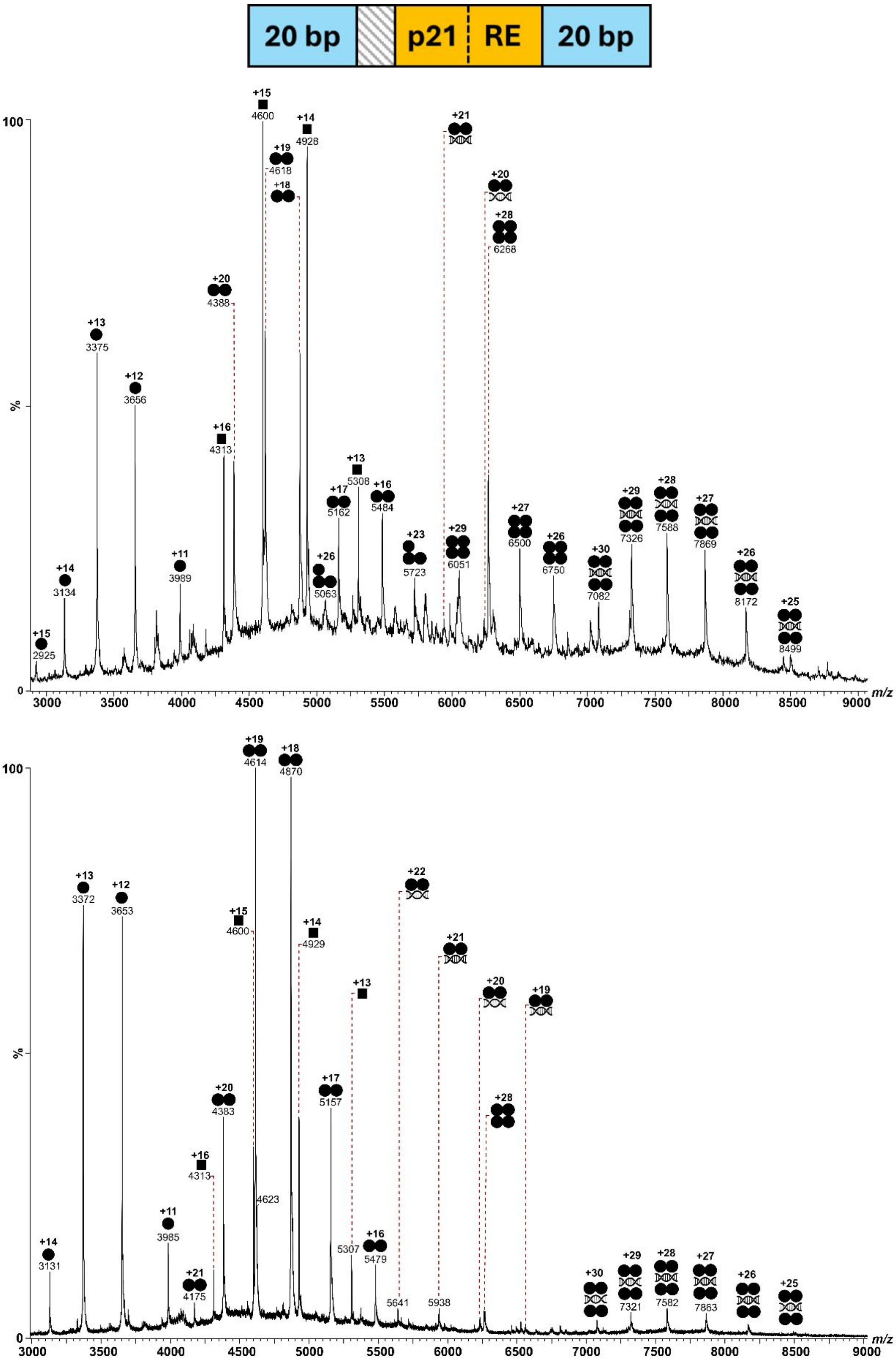

Figure S37. Native mass spectra of $p53_{wild\text{-}type}$ (upper spectrum) and $p53_{L344A}$ (lower spectrum) in the presence of DNA-RE p21_11. The DNA-RE is schematically depicted on top of the mass spectra. p53 (circle), p53:DNA-complex (circle with DNA), DnaK (square), unknown 112 kDa species (triangle), unknown 156 kDa species (pentagon), unknown 199 kDa species (star).
